# Comparative Proteomics and Secretomics Revealed Virulence and Antibiotic Resistance-Associated Factors in *Vibrio parahaemolyticus* Recovered From Commonly Consumed Aquatic Products

**DOI:** 10.3389/fmicb.2020.01453

**Published:** 2020-07-14

**Authors:** Zhuoying Zhu, Lianzhi Yang, Pan Yu, Yongjie Wang, Xu Peng, Lanming Chen

**Affiliations:** ^1^Key Laboratory of Quality and Safety Risk Assessment for Aquatic Products on Storage and Preservation (Shanghai), China Ministry of Agriculture, College of Food Science and Technology, Shanghai Ocean University, Shanghai, China; ^2^Archaea Centre, Department of Biology, University of Copenhagen, Copenhagen, Denmark

**Keywords:** *Vibrio parahaemolyticus*, secretome, proteome, virulence, resistance, aquatic products

## Abstract

*Vibrio parahaemolyticus* is a seafoodborne pathogen that can cause severe gastroenteritis and septicemia diseases in humans and even death. The emergence of multidrug-resistant *V. parahaemolyticus* leads to difficulties and rising costs of medical treatment. The bacterium of environmental origins containing no major virulence genes (*tdh* and *trh*) has been reported to be associated with infectious diarrhea disease as well. Identification of risk factors in *V. parahaemolyticus* is imperative for assuming food safety. In this study, we obtained secretomic and proteomic profiles of *V. parahaemolyticus* isolated from 12 species of commonly consumed aquatic products and identified candidate protein spots by using two-dimensional gel electrophoresis and liquid chromatography tandem mass spectrometry techniques. A total of 11 common and 28 differential extracellular proteins were found from distinct secretomic profiles, including eight virulence-associated proteins: outer membrane channel TolC, maltoporin, elongation factor Tu, enolase, transaldolase, flagellin C, polar flagellin B/D, and superoxide dismutase, as well as five antimicrobial and/or heavy metal resistance-associated ABC transporter proteins. Comparison of proteomic profiles derived from the 12 *V. parahaemolyticus* isolates also revealed five intracellular virulence-related proteins, including aldehyde-alcohol dehydrogenase, outer membrane protein A, alkyl hydroperoxide reductase C, phosphoenolpyruvate-protein phosphotransferase, and phosphoglycerate kinase. Additionally, our data indicated that aquatic product matrices significantly altered proteomic profiles of the *V. parahaemolyticus* isolates with a number of differentially expressed proteins identified. The results in this study meet the increasing need for novel diagnosis candidates of the leading seafoodborne pathogen worldwide.

## Introduction

*Vibrio parahaemolyticus* is a gram-negative bacterium that thrives in marine, estuarine, and aquaculture environments worldwide (Ghenem et al., 2017). The bacterium is frequently detected from seafood such as shellfish, shrimp, and fish. Consumption of raw or inadequately cooked seafood, primarily oysters, can cause acute gastroenteritis in humans (Ghenem et al., 2017). *Vibrio parahaemolyticus* was initially identified in an outbreak of infectious diarrhea disease in 1950 in Osaka, Japan, caused by contaminated semidried juvenile sardines, which sickened 272 and killed 20 people (Fujino et al., 1965). Henceforth, outbreaks and prevalence of the infectious disease are reported in many countries in the world (Ghenem et al., 2017; Baker-Austin et al., 2018). In the United States, there were approximately 35,000 cases of acute gastroenteritis infected by *V. parahaemolyticus* per year from 2000 to 2008 (Baker-Austin et al., 2018). Data from foodborne disease outbreak reporting system in China showed that 42.3% of biohazard cases reported were attributed to *V. parahaemolyticus* from 2011 to 2016 (Liu et al., 2018). *Vibrio parahaemolyticus* has been the leading cause of infectious diarrhea disease, especially among adults in coastal regions in China (Yang C. et al., 2019). Recently, *V. parahaemolyticus* was reported to be associated with acute hepatopancreatic necrosis disease, a newly emerging shrimp disease, which severely damaged the global shrimp industry (Li et al., 2017). The global spread of *V. parahaemolyticus* underscores the need for a better understanding of virulence traits of the bacterium.

Most pathogenic *V. parahaemolyticus* strains have two major virulence genes (*tdh* and *trh*) encoding thermostable direct hemolysin (TDH) and TDH-related hemolysin. The former is a heat-resistant and pore-forming toxin composed of 156 amino acids, whereas the latter contains 189 amino acids and shares 54.8–68.8% identify in nucleotide level with the TDH (Zhang and Orth, 2013). Both these toxins have hemolytic activity, enterotoxin activity, cardiotoxicity, and cytotoxicity (Li et al., 2019). Nevertheless, approximately 90–99% of *V. parahaemolyticus* isolates of environmental origins were detected negative for the toxic factors (Raszl et al., 2016; Park et al., 2018). Previous studies have revealed some other cytotoxic factors to human gastrointestinal cells secreted by type III and type VI secretion systems (T3SS and T6SS) in *V. parahaemolyticus* (Makino et al., 2003). Two types of T3SSs (T3SS1 and T3SS2) were identified, of which T3SS1 located in chromosome I is necessary for the bacterial survival in the environment (Makino et al., 2003; De Nisco et al., 2017). Recent studies indicated that T3SS1 appeared to inject effectors, for example, VopQ, VopR, VopS, and VPA0450, directly into human gastrointestinal cells, which lead to the induction of rapid autophagy followed by cell rounding, eventually cell lysis (Osorio, 2018). The T3SS2 located on chromosome II plays a critical role in the enteropathogenicity of the bacterium (Makino et al., 2003). The effectors of T3SS2 can cause enterotoxicity by destroying the cell cytoskeleton, for example, VopC, VopL, VopV, and VopO, or manipulating cell signaling transduction, for example, VopA, VopT, VopZ, and VPA1380 (De Souza Santos et al., 2017; Osorio, 2018; Luo et al., 2019). Additionally, T6SS is a complex secretory device capable of secreting effectors into host mammalian cells as well as target bacterial cells (Li et al., 2019). It consists of a series of components including structural proteins, translocators, secreted proteins, and some other proteins with auxiliary function, for example, DotU, IcmF, ClpV, Hcp, VgrG, and PAAR (Salomon et al., 2014). Similar to T3SS, *V. parahaemolyticus* encodes two T6SSs (T6SS1 and T6SS2) located on chromosomes I and II, respectively. T6SS1 is active under warm marine-like conditions (3% NaCl, 30°C), whereas T6SS2 is active under low salinity and low temperature (1% NaCl, 23°C) (Salomon et al., 2013). Recent studies have indicated that T6SSs are tightly regulated in pathogenic bacteria, for example, *Vibrio cholerae*, *Pseudomonas aeruginosa*, and *V. parahaemolyticus*, and induced by external conditions and cues such as quorum sensing, salinity, temperature, mucin, chitin, surface sensing, and cell membrane damage (Ben-Yaakov and Salomon, 2019).

There is growing evidence to support that *V. parahaemolyticus* isolates lacking the virulence factors can cause infectious diarrhea disease in humans (Castillo et al., 2018). For example, Thongjun et al. (2013) reported that 9–10% of clinical isolates recovered from diarrhea patients were identified as non-toxigenic *V. parahaemolyticus* in South Thailand between 2001 and 2010. On the other hand, non-toxigenic *V. parahaemolyticus* isolates of environmental origins may possess additional pathogenicity mechanisms. The emergence of multidrug-resistant (MDR) *V. parahaemolyticus* increases the difficulty and cost of clinical treatment (Elmahdi et al., 2016). For instance, Hu and Chen (2016) reported that 74.5% of the *V. parahaemolyticus* strains (*n* = 208) isolated from 10 species of commonly consumed aquatic products in Shanghai, China, were resistant to more than three antimicrobial agents. Yang et al. (2017) isolated 98 *V. parahaemolyticus* strains from 504 seafood samples in 11 provinces of China and found that 68.38% of the isolates showed MDR phenotypes. Recently, Lopatek et al. (2018) reported that 55.8% of the *V. parahaemolyticus* isolates (*n* = 104) recovered from 595 samples collected from Denmark, France, Germany, Italy, the Netherlands, Norway, Poland, Spain, Sri Lanka, and Turkey had resistance to ampicillin and streptomycin and one isolate resistant to ampicillin, streptomycin, and ciprofloxacin. Therefore, identification of virulence and resistance-associated factors in *V. parahaemolyticus* is imperative for food safety systems, particularly in developing countries.

The combination of two-dimensional gel electrophoresis (2D-GE) and liquid chromatography tandem mass spectrometry (LC-MS/MS) techniques is widely used in current proteomics researches (Kim and Cho, 2019). The 2D-GE couples isoelectric focusing (IEF) in the first dimension and sodium dodecyl sulfate–polyacrylamide gel electrophoresis (SDS-PAGE) in the second dimension, which separate proteins according to isoelectric points and molecular masses, respectively. Several hundreds of individual protein abundances separated by 2D-GE can be quantified in the cell population or sample tissues. The LC-MS/MS is also a powerful tool that can efficiently separate and identify constituents in protein mixtures with high sensitivity and specificity (Aslam et al., 2017). For instance, based on the LC-MS/MS technique, Zhong et al. (2019) compared *V. parahaemolyticus* proteomes between the resuscitation state and viable but non-culturable state and found a total of 429 differentially expressed proteins mainly involved in cellular process, establishment of localization, and metabolic process. Pang et al. (2020) analyzed 102 acetylation modified proteins of *Vibrio alginolyticus* HY9901 by LC-MS/MS and identified five virulence factors, including HemL, FabB, FabD, FabF-3, and PhoR.

The People’s Republic of China is the world’s largest producer, consumer, and exporter of aquatic products, including fish, crustaceans, and shellfish (Hu and Chen, 2016). Data from China Fishery Statistical Yearbook (2018) showed that shellfish production (14,371,304 tons) is an important part of maricultural production in China, and the main species include *Ostrea gigas thunberg* (known as oyster) (34.0%), *Ruditapes philippinarum*, *Mactra veneriformis*, and *Paphia undulate* (known as clam) (29.1%), *Placopecten magellanicus* (known as scallop) (14.0%), *Perna viridis* (known as mussel) (6.5%), and *Solen strictus* (known as razor clam) (6.0%). Additionally, *Aristichthys nobilis* (known as bighead carp) and *Ctenopharyngodon idellus* (known as grass carp) are commonly consumed freshwater fish in China, accounting for 18.4% (5,345,641 tons) and 10.7% (3,097,952 tons) of the total freshwater aquaculture production (29,052,930 tons), respectively. The production of *Litopenaeus vannamei* (known as white-leg shrimp) was 1,672,287 tons, which was the most predominant among the crustaceans in 2017 (Zhang et al., 2018). *Oratosquilla oratoria* (known as mantis shrimp) is one of the most economically important species of Pacific shrimp, and its output was 219,087 tons in 2017 (Zhang et al., 2018). Continuous monitoring of food contaminants and identification of risk factors are crucial to safeguard the food supply chain (Chen and Alali, 2018). In our prior researches, *V. parahaemolyticus* contamination in 10 species of aquatic products was surveyed (He et al., 2016; Hu and Chen, 2016). Secretomic profiles of seven *V. parahaemolyticus* strains of two clinical and five aquatic product origins (*Haliotis asinina*, *Moerella iridescens*, *Metapenaeus ensis*, *Exopalaemon carinicauda*, and *L. vannamei*) were compared, and six virulence-associated proteins involved in bacterial pathogenicity were identified (He et al., 2015). Recently, a number of *V. parahaemolyticus* isolates from 25 species of commonly consumed shellfish, crustacean, and fish samples were isolated and characterized (Su et al., unpublished). Among these, 12 *V. parahaemolyticus* isolates recovered from eight shellfish (*M. veneriformis*, *O. gigas thunberg*, *P. undulate*, *P. viridis*, *P. magellanicus*, *R. philippinarum*, *Sinonovacula constricta*, and *S. strictus*), two crustacean (*L. vannamei* and *O. oratoria*), and two fish (*A. nobilis* and *C. idellus*) had multiple resistance to antimicrobial agents and heavy metals. The objectives of this study were (1) to characterize genetic diversity of the *V. parahaemolyticus* isolates recovered from 12 species of aquatic products; (2) to take advantage of 2D-GE and LC-MS/MS technologies to determine secretomic and proteomic profiles of the 12 *V. parahaemolyticus* isolates and to identify common and/or differential extracellular and intracellular proteins by comparative secretomic and proteomic analysis of the isolates from three kinds of aquatic products; and (3) to study implications of various aquatic product matrices on proteomics of the *V. parahaemolyticus* isolates. The secretomes and proteomes between the *V. parahaemolyticus* isolates and pathogenic reference strains were compared as well. To our knowledge, very little of such information in *V. parahaemolyticus* recovered from all the species (except *L. vannamei*) was available to date. The results in this study support the increasing need for novel sites and targets for food safety control of *V. parahaemolyticu*s contamination in aquatic products.

## Materials and Methods

### *V. parahaemolyticus* Strains and Culture Conditions

*Vibrio parahaemolyticus* strains used in this study are listed in Table 1, and their genotypes and phenotypes were characterized by Su et al. (under review). *Vibrio parahaemolyticus* strains were individually inoculated from −80°C stock in our laboratory in Shanghai Ocean University and incubated in tryptic soy broth (TSB) medium (pH 8.5, 3.0% NaCl; Beijing Land Bridge Technology Co., Ltd., Beijing, China) at 37°C. Bacterial cell cultures grown at mid-logarithmic phase with OD_600nm_ values ranging from 0.5 to 0.6 without shaking were collected for extracellular protein extraction, whereas those at late-logarithmic phase with OD_600nm_ values ranging from 0.8 to 1.0 with shaking at 180 revolutions/min (rpm) were used for intracellular protein extraction (He et al., 2015). *Vibrio parahaemolyticus* ATCC33847 (*tdh*^+^ *trh*^–^) and ATCC17802 (*tdh*^–^ *trh*^+^) were used as standard strains (Table 1).

**TABLE 1:** The genotypes and phenotypes of the *V. parahaemolyticus* isolates used in this study.

*V. parahaemolyticus* strain	Source	Year of isolation	*tlh*	*tdh*	*trh*	Resistance to antibiotics	Tolerance to heavy metals
CHN-B2-28	*Ruditapes philippinarum*, China	2017	+	−	−	RIF, STR, AMP	Hg, Zn
CHN-B5-29	*Placopecten magellanicus*, China	2017	+	−	−	RIF, KAN, STR, AMP	Hg, Zn
CHN-B6-62	*Sinonovacula constricta*, China	2017	+	−	−	RIF, KAN, STR, AMP	Hg, Cr, Pb
CHN-B8-26	*Solen strictus*, China	2017	+	−	−	RIF, KAN, STR, AMP	Cu, Hg, Cd, Zn
CHN-N3-2	*Paphia undulate*, China	2017	+	−	−	RIF, TET, STR, AMP	Cu, Hg, Cd, Pb, Zn
CHN-N4-18	*Perna viridis*, China	2017	+	−	−	RIF, KAN, TET, STR, AMP	Cu, Hg, Zn
CHN-N8-5	*Mactra veneriformis*, China	2017	+	−	−	RIF, TET, STR, AMP	Cd
CHN-N10-18	*Ostrea gigas thunberg*, China	2017	+	−	−	RIF, KAN, STR, AMP	Hg, Cd, Zn
CHN-N1-56	*Litopenaeus vannamei*, China	2017	+	−	−	RIF, KAN, TET, STR, AMP	Hg, Cr, Zn
CHN-N2-5	*Oratosquilla oratoria*, China	2017	+	−	−	RIF, KAN, TET, STR, AMP	Hg, Cd
CHN-L7-40	*Aristichthys nobilis*, China	2017	+	−	−	RIF, KAN, STR, AMP	Cu, Cd, Zn
CHN-Q5-1	*Ctenopharyngodon idellus*, China	2017	+	−	−	RIF, KAN, STR, AMP	Hg, Cd, Cr, Zn
ATCC33847*	Gastroenteritis, Maryland, United States	1973	+	+	−	AMP	−
ATCC17802**	Shirasu food poisoning, Japan	1965	+	−	+	−	−

RIF, rifampin; AMP, ampicillin; STR, streptomycin; KAN, kanamycin; TET, tetracycline; copper, CuCl_2_; mercury, HgCl_2_; cadmium, CdCl_2_; lead, PbCl_2_; zinc, ZnCl_2_; chromium, CrCl_3_; +, present; −, absence. *Baumann et al. (1973); **Fujino et al. (1965).

### Enterobacterial Repetitive Intergenic Consensus—Polymerase Chain Reaction Assay

Bacterial genomic DNA was extracted by a thermal lysis method as previously described (Xu et al., 2019). The Enterobacterial Repetitive Intergenic Consensus (ERIC) primers ERIC-F (5′-ATGTAAGCTCCTGGGGATTCAC-3′) and ERIC-R (5′-AAGTAAGTGACTGGGGTGAGCG-3′) (Rivera et al., 1995) were synthesized by Sangon Biotech Co., Ltd., Shanghai, China. A 20 μL of ERIC–polymerase chain reaction (PCR) reaction solution contained 6 μL sterile DNase/RNase-free deionized H_2_O (Tiangen Biotech Co., Ltd., Beijing, China), 1 μL of each primer (0.25 mM), 10 μL 2 × Taq PCR master mix (Novoprotein Scientific lnc., Shanghai, China), and 2 μL DNA template. The ERIC-PCRs was performed using Mastercycler^®^ pro PCR thermal cycler (Eppendorf Corporate, Hamburg, Germany) under the following conditions: denaturation at 95°C for 8 min, 32 cycles of 95°C for 30 s, 52°C for 1 min, and 65°C for 8 min and a final extension of 65°C for 16 min. The amplified products were analyzed by electrophoresis with 1.0% (wt/vol) agarose gels [Biowest Agarose, Spain (Origin), distributed by Shanghai Fushen Bio-Technology Co., LTD., Shanghai, China] and imaged using UVP EC3 Imaging system (UVP LLC, Upland, CA, United States). Fingerprint patterns were analyzed using BioNumerics 7.6 software, and the clustering was deduced based on the unweighted pair group with arithmetic average (UPGMA) algorithm (Xu et al., 2019).

### Extraction of Extracellular and Intracellular Proteins of *V. parahaemolyticus* Isolates

Extracellular proteins of *V. parahaemolyticus* isolates were extracted according to the method described previously (He et al., 2015) with minor modifications. Briefly, growth curves of *V. parahaemolyticus* isolates incubated in the TSB medium (pH 8.5, 3% NaCl) at 37°C were determined using Bioscreen Automatic Growth Curve Analyzer (BioTek Instruments, Inc., Winooski, VT, United States). A 20 μL of Bacterial Protease Inhibitors Complex (Sangon) was added to each 100 mL of bacterial cell culture at midlogarithmic phase, which was then centrifuged at 6,000 *g* for 10 min at 4°C. The supernatant was filtered through 0.22-μm-pore-size membrane filters (Millipore, Bedford, MA, United States) to remove residual bacterial cells. The filtrate was precipitated by adding ice-cold trichloracetic acid (Sinopharm Chemical Reagent Co., Ltd., Shanghai, China) to a final concentration of 10% (vol/vol) and then incubated on ice for 5 h. The extracellular proteins were collected by centrifugation at 12,000 rpm for 30 min at 4°C. The resulting protein pellet was washed with ice-cold acetone (Sinopharm) for three times, air-dried and stored at −80°C for further analysis.

For the extraction of intracellular proteins, *V. parahaemolyticus* isolates were individually incubated in the TSB medium (pH 8.5, 3.0% NaCl) to late-logarithmic phase at 37°C with shaking. Bacterial cell cultures were collected as described above, and intracellular proteins were extracted using the Bacterial Protein Extraction Kit (Sangon) containing protease inhibitors, according to the manufacturer’s instructions. Protein concentration was measured using Bradford Protein Assay Kit (Sangon) with bovine serum albumin as the standard protein.

### The 2D-GE Assay

The 2D-GE was performed according to the method described previously (He et al., 2015) with minor modifications. Briefly, extracellular protein pellet was dissolved with 100 μL of lysis buffer [8 M urea (Sangon), 4% (wt/vol) 3-[3-cholamidopropyldimethylammonio-1-propanesulfonate (CHAPS; Sangon), 65 mM dithiothreitol (DTT; Sangon), 0and.2% (vol/vol) Bio-Lyte 3/10 ampholyte (Bio-Rad Laboratories Inc., Hercules, CA, United States)] and then centrifuged at 12,000 rpm for 10 min at room temperature to remove undissolved residues. Isoelectric focusing was performed with the ready immobilized pH gradient gel (IPG) strips (Bio-Rad). Approximately 20 μg of extracellular proteins was diluted with the rehydration buffer [8 M urea, 4% (wt/vol) CHAPS, 65 mM DTT, 0.2% (vol/vol) Bio-Lyte 3/10 ampholyte, and 0.0001% (wt/vol) bromophenol blue (Sangon)] to a final volume of 200 μL per sample. The mixture of each sample was applied to the strips (pH 4–7, 7 cm; Bio-Rad) and passive rehydrated for 16 h at 17°C. After rehydration, IEF was run with a 6-step program: 100 V for 3 h with slow ramping; 250 V for 1 h with slow ramping; 500 V for 1 h with linear ramping; 4,000 V for 3 h with linear ramping; and 4,000 V with rapid ramping until 20,000 V-hour was reached. Following the electrophoresis in the first dimension, the strips were first equilibrated for 15 min in equilibration buffer I [6 M urea, 37.5 mM Tris-HCl (pH 8.8, Sangon), 20% glycerol (Sangon), 2% SDS (Sangon), and 2% DTT] and then washed for another 15 min with equilibration buffer II [6 M urea, 37.5 mM Tris-HCl at pH 8.8, 2% SDS, 20% glycerol, 2.5% (wt/vol) iodoacetamide (Sangon)]. The second-dimension separation was performed by SDS-PAGE. The strips were individually transferred onto 12.5% separation gel using a Mini-PROTEANW electrophoresis cell (Bio-Rad) with a 2-step program: 5 mA for 20 min, and 15 mA for 85 min.

For intracellular proteins, an aliquot of each 400 μL of protein sample was individually applied to the strips (pH 4–7, 17 cm) (Bio-Rad) and passive rehydrated for 17 h at 17°C. After rehydration, IEF was run with a 9-step program: 50, 100, 500, and 1,000 V for 1 h with slow ramping, respectively; 2,000 and 4,000 V for 1 h with linear ramping, respectively; 6,000 and 8,000 V for 1 h with rapid ramping, respectively; and 10,000 V with rapid ramping until 80,000 V-hour was reached. The strips were individually transferred onto 12.5% separation gel using PROTEAN II XL electrophoresis cell (Bio-Rad) with a 2-step program: 5 mA for 5 h and 15 mA for 6 h.

After electrophoresis, the gels were stained using Protein Stains K (Sangon), according to the manufacturer’s instructions. Silver-stained gels were scanned using GenoSens 1800 Series Gel Documentation and Analysis System (Clinx Science Instruments Co. Ltd., Shanghai, China). Protein spot detection, spot matching, and quantitative intensity analysis were performed using PDQuest Advanced-8.0.1 software (Bio-Rad).

### LC-MS/MS Analysis

The LC-MS/MS analysis was carried out at HooGen Biotech, Shanghai, China. The visible and discriminative protein spots were individually excised from 2D-GE gels, and gel pieces were dried under vacuum. A 15 μL of digestion solution (25 mM ammonium bicarbonate) containing trypsin (12.5 ng/mL; Promega, Madison, WI, United States) was added to each protein spot sample tube and incubated with gentle shaking at 37°C for 16 h. The resulting peptides were identified by Q Exactive Mass Spectrometer [Thermo Fisher Scientific (TFS), Waltham, MA, United States] coupled with Easy nLC 1200 Chromatography System (TFS). The peptide mixture was loaded onto C18-reversed phase column (15 cm long, 75-μm inner diameter) packed in-house with RP-C18 5 μm resin in buffer A [0.1% formic acid (Sigma-Aldrich, St. Louis, MO, United States) in high-performance liquid chromatography–grade water] and separated with a linear gradient of buffer B [0.1% formic acid in 84% acetonitrile (Sigma-Aldrich, United States)] at a flow rate of 250 nL/min over 60 min. The collected LC-MS/MS data files were converted to Mascot generic format (mgf) and then imported into Mascot version 2.2 server (Matrix Science, London, United Kingdom) for automated peptide identification using UniProt *Vibrio parahaemolyticus* database (download in September 2019; 89189 sequences). The criteria for peptide matching and protein calls included enzyme: trypsin; max missed cleavage: 2; fixed modifications: carbamidomethyl (C); variable modification: oxidation (M); peptide mass tolerance: ± 20 ppm; fragment MS/MS tolerance: 0.1 Da; Mascot score: ≥20; ion score: >20; and false discovery rate: <0.01 at peptide and protein level. Common protein spots on secretomic profiles were marked with blue circles, whereas differential protein spots on secretomic and proteomic profiles were marked in red numbers. The identified proteins were subjected to Generic Gene Ontology (GO) Term Finder^1^ for GO analysis (Yang et al., 2015).

### Preparation of Aquatic Product Matrix Media

Aquatic product matrices were prepared according to the method described by Chaitiemwong et al. (2010) with minor modifications. Briefly, 12 species of fresh aquatic products were collected in two local fish markets [Guzong road fish market (30°53′11.34″N, 121°55′3.09″E) and Luchaogang seafood market (30°51′34.47″N, 121°51′3.15″E)] in Shanghai and transferred in sterile sealed plastic bags (Nanjing Maojie Microbial Technology Co., Ltd., Nanjing, China) in an ice box (700 × 440 × 390 mm) to the laboratory in Shanghai Ocean University for analysis immediately. A 10 g (wet weight) fresh meat of each sample was aseptically placed in a homogeneous bag (Sangon) with 40 mL sterile water and homogenized for 3 min using a laboratory blender BagMixer (Shanghai Jingxin Industry Development Co., Ltd., Shanghai, China). The mixture was centrifuged at 8,000 *g* for 20 min, and the supernatant was filtered through 0.22-μm-pore-size membrane filters (Millipore). The filtrate of each aquatic product sample was supplemented into the TSB medium (pH 8.5, 3% NaCl) to produce corresponding aquatic product matrix medium with a final concentration of 1% (vol/vol).

### Measurement of Compositions of Aquatic Product Matrices

Total protein content was measured using Bradford Protein Assay Kit (Sangon) according to the manufacturer’s instructions. Crude fat content was determined by the automated Soxhlet method described by Shin et al. (2013) with minor modifications. Briefly, a 5 mL of aquatic product matrix was individually weighed and dried in a Petri dish at 104°C for 3 h. The dried products were wiped with cotton balls moistened with petroleum ether (Shanghai Titan Scientific Co., Ltd., Shanghai, China). Crude fat on cotton balls was extracted by an automated Soxtherm Fat Extraction System (Gerhardt, Bonn, Germany) according to the manufacturer’s instructions. The extracted products remaining in a weighed beaker were dried at 104°C for 30 min and cooled in a dessicator to room temperature. Crude fat in the beakers was weighed and calculated. Carbohydrate (saccharides) content was measured by the phenol–sulfuric acid method described by Albalasmeh et al. (2013) with minor modifications. A 1 mL of each sample was mixed with 1 mL of 5% (wt/wt) aqueous solution of redistilled phenol (Shanghai Macklin Biochemical Co., Ltd., Shanghai, China) in a glass tube (Φ15 mm, 150 mm). A 5 mL of sulfuric acid (95–98%; Shanghai Kuling Fine Chemical Co., Ltd., Shanghai, China) was added into the mixture and then incubated in a boiling water bath for 20 min. After cooling in ice for 2 min and at room temperature for 15 min, the absorption of the reaction mixture at 490 nm was measured using BioTek Synergy 2. Carbohydrate concentration was calculated according to a standard curve prepared with glucose (Sinopharm).

### Reverse Transcription–PCR Assay

The reverse transcription (RT)–PCR assay was performed according to the method described by Zhu et al. (2017). Briefly, total RNA was extracted using RNeasy Mini Kit (Qiagen, Hilden, Germany) following the manufacturer’s instructions. The DNA was removed from the samples with the RNase-Free DNase Set (Qiagen). The RT reactions were performed using PrimeScript^TM^ RT reagent Kit with gDNA Eraser [Takara Biomedical Technology (Beijing) Co., Ltd., Beijing, China]. Relative quantitative PCRs were performed using TB Green^®^ Premix Ex Taq^TM^ II kit (Tli RNaseH Plus, Beijing, China) according to the manufacturer’s instructions. All RT-PCRs were carried out in a 7500 Fast Real-Time PCR System (Applied Biosystems, Foster City, CA, United States) under the following conditions: initial denaturation at 50°C for 2 min and at 95°C for 10 min, followed by 40 cycles of denaturation at 95°C for 15 s and primer annealing at 60°C for 1 min. The 16S RNA was used as the internal reference gene as described previously. The primers (Supplementary Table S1) were designed by Premier 5.0 software^2^ and synthesized by Sangon (Shanghai, China). In a 20 μL of RT-PCR solution, 1,000, 200, 20, 2, 0.2, and 0.0 ng of cDNA template was individually added for standard curve preparation. The PCR efficiency was 90.0–100.0%, and *r*^2^ > 0.980 for the primers. All tests were performed in triplicate in this study.

## Results

### Genetic Diversity of the *V. parahaemolyticus* Isolates Recovered From 12 Species of Aquatic Products

The *V. parahaemolyticus* isolates used in this study were recovered from 12 species of commonly consumed aquatic products, including eight shellfish, two crustaceans, and two fish (Table 1). All the isolates were negative for the toxin genes (*tdh* and *trh*), but resistant to three antimicrobial agents, including rifampin (RIF), ampicillin (AMP), and streptomycin (STR). Approximately 75% (9/12) and 42% (5/12) of the *V. parahaemolyticus* isolates were also resistant to kanamycin (KAN) and tetracycline (TET), respectively. Additionally, different heavy metal tolerance profiles of the *V. parahaemolyticus* isolates were observed. Remarkably, *V. parahaemolyticus* CHN-N3-2 isolate recovered from the shellfish *P. undulate* had tolerance to five heavy metals Cd^2+^, Cu^2+^, Hg^2+^, Pb^2+^, and Zn^2+^. The heavy metal tolerance trend of the 12 *V. parahaemolyticus* isolates was Hg^2+^ = Zn^2+^ > Cd^2+^ > Cu^2+^ > Cr^3+^ > Pb^2+^ (Table 1).

**FIGURE 1:**
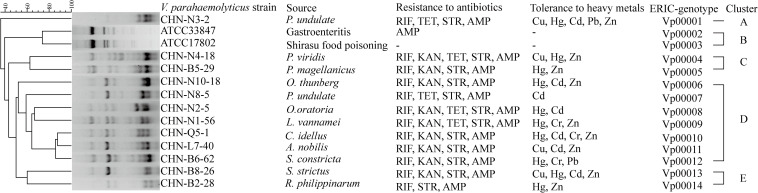
The ERIC-PCR fingerprinting profiles of the *V. parahaemolyticus* strains.

Evolutionary relatedness of the *V. parahaemolyticus* strains was examined by the ERIC-PCR assay. This analysis revealed that all the tested strains were classified into 14 different ERIC genotypes, and the UPGMA algorithm grouped the ERIC genotypes into five distinct clusters (Clusters A–E) (Figure 1). *Vibrio parahaemolyticus* ATCC33847 (*tdh*^+^ *trh*^–^) and ATCC17802 (*tdh*^–^ *trh*^+^) strains of clinical origin fell into Cluster B, whereas the remaining of aquatic product origins fell into Clusters A, C, D, and E. *Vibrio parahaemolyticus* isolates recovered from three shellfish (*O. gigas thunberg*, *P. undulate*, and *S. constricta*), two crustaceans (*L. vannamei* and *O. oratoria*), and two fish (*A. nobilis* and *C. idellus*) were grouped into the largest Cluster D with 7 ERIC genotypes, whereas Cluster A contained only the *V. parahaemolyticus* CHN-N3-2 isolate that had tolerance to 4 antimicrobial agents and 5 heavy metals. The Hg and AMP/KAN/RIF/STR resistance profile was the most predominant among the strains tested (Figure 1). These results demonstrated genetic diversity of the *V. parahaemolyticus* isolates recovered from the 12 species of commonly consumed aquatic products.

### Distinct Secretomic Profiles of the *V. parahaemolyticus* Isolates

**FIGURE 2:**
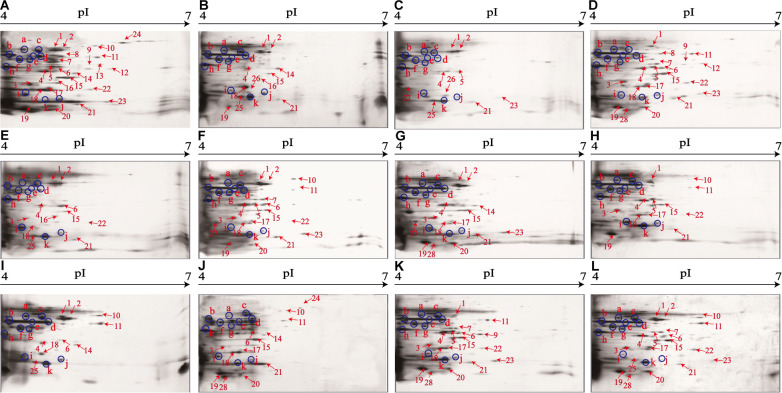
The secretomic profiles of the 12 *V. parahaemolyticus* isolates by the 2D-GE analysis. **(A–L)** *V. parahaemolyticus* CHN-B2-28, CHN-B5-29, CHN-B6-62, CHN-B8-26, CHN-N3-2, CHN-N4-18, CHN-N8-5, CHN-N10-18, CHN-N1-56, CHN-N2-5, CHN-L7-40, and CHN-Q5-1 isolates, respectively. The common protein spots marked with blue circles and the differential protein spots marked with different numbers in red were characterized by the LC-MS/MS analysis. pI, isoelectric point.

The supernatant of the *V. parahaemolyticus* cultures at midlogarithmic phases was collected, and extracellular proteins were extracted and analyzed by 2D-GE. This analysis uncovered distinct secretomic profiles of the 12 *V. parahaemolyticus* isolates of various aquatic product origins (Figure 2). The patterns yielded from three independent 2D-GE gels of each isolate were consistent (figures not shown). Based on the consensus patterns, 11 extracellular protein spots (marked with blue circles, Figure 2) were observed at similar locations on all the 2D-GE patterns derived from the 12 *V. parahaemolyticus* isolates. Notably, the *V. parahaemolyticus* CHN-B2-28 isolate recovered from *R. philippinarum* appeared to secret more extracellular proteins (35) than the other isolates (17–30). Additionally, a total of 28 differential extracellular proteins (marked with different numbers in red, Figure 2) were found from the distinct secretomic profiles. All common protein spots and the remaining differential protein spots among the 12 *V. parahaemolyticus* isolates were excised from the 2D-GE gels and digested with the trypsin. The resulting peptides were further identified by the LC-MS/MS analysis.

### Identification of Common and Differential Extracellular Proteins of the *V. parahaemolyticus* Isolates

In this study, the LC-MS/MS technique was used to identify all common and differential extracellular proteins of the *V. parahaemolyticus* isolates. Based on the secretomic profiles, 11 common extracellular proteins shared among the 12 isolates were successfully obtained (Table 2). Of these proteins, the majority (*n* = 7) were cell membrane and periplasm components, including TolC protein (Spot a), maltoporin (Spot b), maltodextrin-binding protein (Spot d), putrescine-binding periplasmic protein (Spot f), gram-negative porin family protein (Spot h), outer membrane protein W (OmpW, Spot j), and basal-body rod modification protein FlgD (Spot i). For the others (*n* = 4), the Spot c was identified as elongation factor Tu (EF-Tu). Spot e was phosphoglucosamine mutase (PNGM) involved in carbohydrate metabolism and peptidoglycan biosynthetic pathway, whereas Spot g was fructose-bisphosphate aldolase that also acts as a transcriptional regulator in pathogenic *Francisella* (Ziveri et al., 2017). Additionally, the Spot k matched an uncharacterized protein encoded by the *H320_01320* gene of *V. parahaemolyticus* 49 strain (GenBank: AONA00000000.1) with currently unknown function in public databases (Table 2).

**TABLE 2:** Identification of the common protein spots on the secretomic profiles of the 12 *V. parahaemolyticus* isolates by LC-MS/MS analysis.

Protein spot no.	Uniprot no.	Protein	Gene	MW (Da)	pI	Sequence coverage (%)	Putative function
a	A6B7I5	TolC protein	*A79_3452*	47,982.46	4.72	32.13	Efflux transmembrane transporter activity, outer membrane
b	A0A2R9VS99	Maltoporin	*lamB*	46,237.27	4.54	29.55	Maltodextrin transmembrane transporter activity, maltose transporting porin activity, ion transport, cell outer membrane
c	A0A0M9CAT1	Elongation factor Tu	*tuf*	43,151.54	4.8	11.17	GTPase activity, GTP binding, translation elongation factor activity, cytoplasm
d	S5IUW0	Maltodextrin-binding protein	*malE*	42,126.25	4.84	41.07	Carbohydrate transmembrane transporter activity, periplasm
e	Z2EVN8	Phosphoglucosamine mutase	*glmM*	39,259.29	4.74	14.17	Magnesium ion binding, phosphoglucosamine mutase activity, carbohydrate metabolic process
f	A0A0D1FMZ9	Putrescine-binding periplasmic protein	*potD*	39,016.78	4.63	29.57	Polyamine binding, polyamine transport, periplasm
g	S5ISY4	Fructose-bisphosphate aldolase	*M634_15410*	38,880.23	4.7	11.45	Fructose-bisphosphate aldolase activity, zinc ion binding, glycolytic process
h	Z2EPJ8	Gram-negative porin family protein	*D046_2206*	36,284.98	4.25	18.69	Porin activity, membrane
i	Q79YY9	Basal-body rod modification protein FlgD	*VP0777*	24,895.75	4.75	54.47	Bacterial-type flagellum organization, cell projection, cilium, flagellum
j	S5J784	Outer membrane protein W	*M634_18560*	23,467.38	4.98	14.02	Outer membrane
k	A0A0D1ER15	Uncharacterized protein	*H320_01320*	19,780.83	4.93	29.83	—*

*—, not detected.

A total of 28 differential extracellular proteins were also successfully identified and listed in Table 3. The metabolism-related proteins (*n* = 7) constituted the largest proportion of the identified proteins, including three glycolysis-related proteins: enolase (Spot S2), phosphofructokinase (PFK) (Spot S6), and glyceraldehyde-3-phosphate dehydrogenase (GAPDH, Spot S13); one carbohydrate metabolism-related protein: transaldolase (Spot S5); one protein catabolism-related protein: ATP-dependent zinc metalloprotease (FtsH, Spot S10); one tricarboxylic acid cycle-related protein: succinate dehydrogenase iron–sulfur subunit (SDISS, Spot S17); and one polyphosphate metabolism-related protein: inorganic pyrophosphatase (PPase, Spot S25). Moreover, five differential extracellular proteins were involved in amino acid and nucleotide biosynthesis. For example, Spots S3, S11, and S22 were identified as 4-hydroxy-tetrahydrodipicolinate synthase (DHDPS), aspartate-semialdehyde dehydrogenase (ASD), and elongation factor Ts (EF-Ts) in amino acid biosynthesis, respectively, whereas Spots S1 and S9 were adenylosuccinate synthetase (AdSS) and ribose-phosphate pyrophosphokinase in nucleotide biosynthesis, respectively. Notably, two bacterial flagellin structural proteins were identified, including flagellin C (Spot S7) and polar flagellin B/D (Spot S8). In addition, Spot S27 matched an uncharacterized protein encoded by the *E4P16_05165* gene of *V. parahaemolyticus* MAVP-R strain (GenBank: CP022553.2).

**TABLE 3:** Identification of the differential protein spots on the secretomic profiles of the 12 *V. parahaemolyticus* isolates by LC-MS/MS analysis.

Protein spot no.	Uniprot no.	Protein	Gene	MW (Da)	pI	Sequence coverage (%)	Putative function
S1	A0A0D8WTD1	Adenylosuccinate synthetase	*purA*	45,652.88	4.83	12.44	Adenylosuccinate synthase activity, GTP binding, magnesium ion binding, AMP biosynthetic process, cytoplasm
S2	Z2ECU7	Enolase	*eno*	45,561.11	4.84	25.64	Magnesium ion binding, phosphopyruvate hydratase activity, glycolytic process, cytoplasm, secreted, cell surface, extracellular region, phosphopyruvate hydratase complex
S3	A0A0M9C0M8	4-Hydroxy-tetrahydrodipicolinate synthase	*dapA*	31,256.2	4.71	22.26	4-Hydroxy-tetrahydrodipicolinate synthase activity, diaminopimelate biosynthetic process, lysine biosynthetic process via diaminopimelate, cytoplasm
S4	A0A0D1E7V7	Thioredoxin reductase	*H320_01355*	34,458.4	4.8	19.75	Thioredoxin-disulfide reductase activity, removal of superoxide radicals, cytoplasm
S5	A0A0D1EJC9	Transaldolase	*tal*	34,805.45	4.86	17.09	Sedoheptulose-7-phosphate: D-glyceraldehyde-3-phosphate glyceronetransferase activity, carbohydrate metabolic process, pentose-phosphate shunt, cytoplasm
S6	S5ITG7	Phosphofructokinase	*fruK*	34,822.23	4.99	9.88	1-Phosphofructokinase activity, ATP binding
S7	A0A0D1EZB6	Flagellin C	*H334_00575*	39,848.5	4.86	39.63	Structural molecule activity, bacterial-type flagellum-dependent cell motility
S8	Q56702	Polar flagellin B/D	*flaB*	40,172.91	4.89	43.12	Structural molecule activity, bacterial-type flagellum-dependent cell motility
S9	A0A0M9CAE5	Ribose-phosphate pyrophosphokinase	*prs*	33,916.46	5.16	12.74	ATP binding, kinase activity, magnesium ion binding, ribose phosphate diphosphokinase activity, 5-phosphoribose 1-diphosphate biosynthetic process, nucleoside metabolic process, nucleotide biosynthetic process, ribonucleoside monophosphate biosynthetic process
S10	A0A0D1UWT7	ATP-dependent zinc metalloprotease FtsH	*hflB*	72,648.12	5.11	5.61	ATPase activity, ATP binding, metalloendopeptidase activity, zinc ion binding, protein catabolic process
S11	A0A072K9I6	Aspartate-semialdehyde dehydrogenase	*asd*	40,210.86	5.29	4.58	Aspartate-semialdehyde dehydrogenase activity, NADP binding, *de novo* L-methionine biosynthetic process, diaminopimelate biosynthetic process, lysine biosynthetic process via diaminopimelate, threonine biosynthetic process
S12	A0A0L8RX62	Glycine/betaine ABC TSBP	*WR32_14495*	34,236.49	5.6	24.36	Choline binding, transmembrane transporter activity, choline transport, ATP-binding cassette (ABC) transporter complex, periplasmic space
S13	Z2F2I8	Glyceraldehyde-3-phosphate dehydrogenase	*D046_0544*	35,225.62	5.26	25.38	NAD binding, NADP binding, oxidoreductase activity, acting on the aldehyde or oxo group of donors, NAD or NADP as acceptor
S14	A0A4Q9JZC8	Choline ABC TSBP	*D5E78_23820*	34,357.45	5.06	33.33	Choline binding, transmembrane transporter activity, periplasm
S15	A0A0D1FHL7	D-Ribose ABC TSBP	*H323_16900*	30,690.84	4.98	62.67	Hydrolase activity, kinase activity
S16	A6AZB7	D-Ribose-binding periplasmic protein	*A79_4530*	30,676.77	4.91	65.07	ATPase-coupled monosaccharide transmembrane transporter activity, hydrolase activity
S17	A0A0D1F2I1	Succinate dehydrogenase iron–sulfur subunit	*H323_04040*	27,500.27	4.94	31.30	2 Iron, 2 sulfur cluster binding, 3 iron, 4 sulfur cluster binding, 4 iron, 4 sulfur cluster binding, electron transfer activity, metal ion binding, succinate dehydrogenase (ubiquinone) activity, tricarboxylic acid cycle
S18	A0A0L7Z6L0	Arginine ABC TSBP	*ACS91_16450*	27,341.39	4.82	10.93	Ligand-gated ion channel activity, nitrogen compound transport, membrane, outer membrane-bounded periplasmic space
S19	A0A0L7YZR8	Cytochrome C	*ACS91_10120*	14,421.19	4.57	25.55	Electron transfer activity, heme binding, iron ion binding
S20	A0A0D1ELJ0	L-Ectoine synthase	*ectC*	14,752.49	4.94	18.75	Ectoine synthase activity
S21	A0A0D1G7K7	DNA starvation/stationary phase protection protein	*H320_14130*	18,275.43	5.07	22.01	Ferric iron binding, oxidoreductase activity, oxidizing metal ions, cellular iron ion homeostasis, cell
S22	Q87MD9	Elongation factor Ts	*tsf*	29,773.89	5.18	13.17	Translation elongation factor activity, cytoplasm
S23	A0A2R9VKF8	Outer membrane lipoprotein carrier protein	*lolA*	23,437.09	5.37	22.60	Lipoprotein localization to outer membrane, lipoprotein transport, periplasmic
S24	Q87TM1	Peptide ABC TPPBP	*VP0048*	57,448.81	5.96	4.45	Transmembrane transport
S25	Z2ED59	Inorganic pyrophosphatase	*ppa*	19,644.14	4.82	48.30	Inorganic diphosphatase activity, magnesium ion binding, phosphate-containing compound metabolic process, cytoplasm
S26	A0A072IV06	Superoxide dismutase	*sodB*	21,540.77	4.95	13.40	Metal ion binding, superoxide dismutase activity
S27	A0A4S3TGP2	Uncharacterized protein	*E4P16_05165*	31,030.6	4.65	16.78	—*
S28	A0A072LHX1	Nitrogen regulatory protein P-II	*ACS91_04985*	12,471.24	4.87	42.86	Enzyme regulator activity, regulation of nitrogen utilization

*—, not detected.

Interestingly, five differential extracellular proteins were identified as ATP-binding cassette (ABC) transporters that constitute one of the largest families of membrane proteins (Theodoulou and Kerr, 2015), including glycine/betaine ABC transporter substrate-binding protein (TSBP) (Spot S12), choline ABC TSBP (Spot S14), D-ribose ABC TSBP (Spot S15), arginine ABC TSBP (Spot S18), and peptide ABC transporter periplasmic peptide-binding protein (TPPBP) (Spot S24). Moreover, the Spot S16 was D-ribose-binding periplasmic protein that has ATPase-coupled monosaccharide transmembrane transporter activity, whereas Spot S23 matched outer membrane lipoprotein carrier protein.

Remarkably, in this study, several identified extracellular proteins of the *V. parahaemolyticus* isolates have been reported to be involved in bacterial pathogenesis, including Spots a, b, c, S2, S5, S7, S8, and S26. The former three proteins TolC (Spot a), maltoporin (Spot b), and EF-Tu (Spot c) were secreted by all the isolates, and more than half of the isolates also secreted the enolase (Spot S2) and transaldolase (Spot S5). In contrast, only a few isolates secreted the flagellin C (Spot S7), polar flagellin B/D (Spot S8), and superoxide dismutase (Spot S26). For instance, the latter was only observed on the secretomic profiles derived from the CHN-B5-29 and CHN-B6-62 isolates recovered from *P. magellanicus* and *S. constricta*, respectively. Among all the *V. parahaemolyticus* isolates of aquatic product origins, the CHN-B2-28 isolate from *R. philippinarum* appeared to secret most of the extracellular virulence-associated proteins compared to the other isolates.

### Secretomic Comparison of the *V. parahaemolyticus* Isolates From Three Kinds of Aquatic Products

The secretomic profiles derived from the *V. parahaemolyticus* isolates of the shellfish, crustaceans, and fish origins were different. For instance, there were 14 common and 14 differential extracellular proteins identified from the secretomic profiles of the *V. parahaemolyticus* CHN-B2-28, CHN-B5-29, CHN-B6-62, CHN-B8-26, CHN-N3-2, CHN-N4-18, CHN-N8-5, and CHN-N10-18 isolates recovered from the eight species of shellfish (Figures 2A–H). Significantly, there were six extracellular proteins appeared only on the secretomic profile of the shellfish origin, including the polar flagellin B/D (Spot S8), glycine/betaine ABC TSBP (Spot S12), GAPDH (Spot S13), D-ribose-binding periplasmic protein (Spot S16), superoxide dismutase (Spot S26), and an uncharacterized protein (Spot S27) (Table 3).

Comparison of the secretomic profiles of the *V. parahaemolyticus* isolates from two crustaceans revealed that the *V. parahaemolyticus* CHN-N2-5 isolate recovered from *L. vannamei* secreted more abundant proteins than the CHN-N1-56 isolate from *O. oratoria* (Figures 2I,J). A total of 16 common and 12 differential extracellular proteins on the secretomic profiles of these two isolates were identified. Spots S1, S2, S4, and S25, identified as AdSS, enolase, thioredoxin reductase (TrxR), and PPase, respectively, were secreted only by the CHN-N1-56 isolate, whereas eight proteins were secreted by the CHN-N2-5 isolate, including the DHDPS (Spot S3), D-ribose ABC TSBP (Spot S15), SDISS (Spot S17), cytochrome C (Spot S19), L-ectoine synthase (Spot S20), DNA starvation/stationary phase protection protein (Spot S21), peptide ABC TPPBP (Spot S24), and nitrogen regulatory protein P-II (Spot S28).

The numbers of extracellular proteins secreted by the *V. parahaemolyticus* CHN-L7-40 and CHN-Q5-1 isolates of the fish origin were similar. A total of 26 common and five differential extracellular proteins on the secretomic profiles of these two isolates were identified. For instance, Spots S9 and S18, identified as ribose-phosphate pyrophosphokinase and arginine ABC TSBP, respectively, were shown only on the secretomic profile of the CHN-L7-40 isolate from *A. nobilis* (Figure 2K), whereas Spots S2, S10, and S25, identified as enolase, ATP-dependent zinc metalloprotease FtsH, and PPase, respectively, were secreted only by the CHN-Q5-1 isolate from *C. idellus* (Figure 2L).

### Secretomic Comparison of the *V. parahaemolyticus* Isolates With Pathogenic Reference Strains

The secretomic profiles of the *V. parahaemolyticus* ATCC33847 and ATCC17802 strains of clinical origin were also obtained for comparative secretomic analysis, although they have been reported in our prior research (He et al., 2015). One interesting observation was that the toxic ATCC33847 and ATCC17802 strains appeared to secret more extracellular proteins (59–62) than the *V. parahaemolyticus* isolates recovered from the 12 species of aquatic products (17–35), consistent with our prior results (He et al., 2015). Comparative secretomic analysis revealed that approximately 36.4% (4/11) of the common and 42.9% (12/28) of the differential extracellular proteins secreted by the 12 *V. parahaemolyticus* isolates were observed at similar locations on the secretomic profiles of the ATCC33847 and the ATCC17802 strains (Supplementary Figure S1). For instance, four extracellular proteins secreted by all the *V. parahaemolyticus* strains of aquatic product and clinical origins included the maltoporin (Spot b), EF-Tu (Spot c), maltodextrin-binding protein (Spot d), and PNGM (Spot e).

### Distinct Proteomic Profiles of the *V. parahaemolyticus* Isolates

**FIGURE 3:**
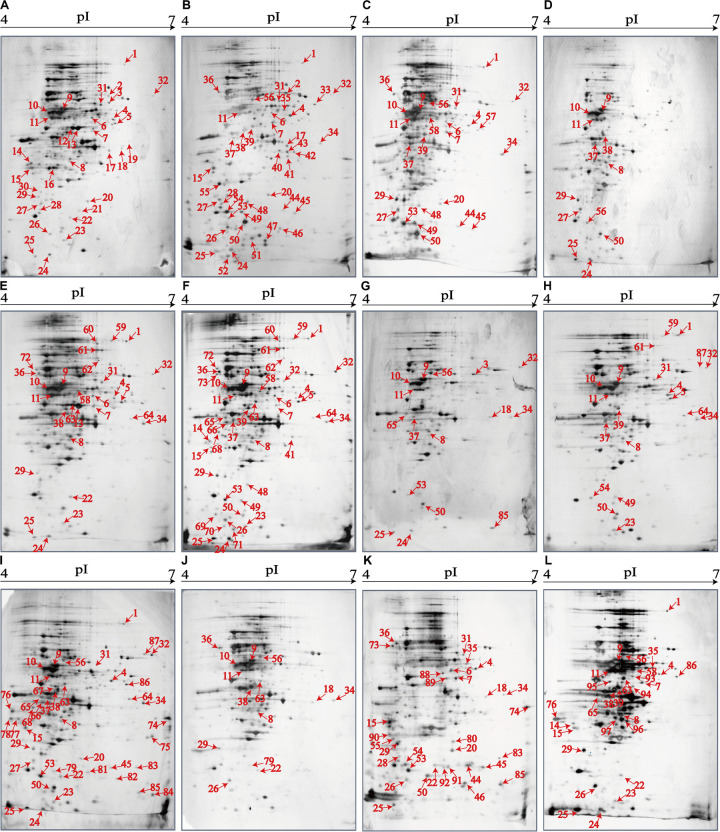
The proteomic profiles of the 12 *V. parahaemolyticus* isolates by the 2D-GE analysis. **(A–L)** *V. parahaemolyticus* CHN-B2-28, CHN-B5-29, CHN-B6-62, CHN-B8-26, CHN-N3-2, CHN-N4-18, CHN-N8-5, CHN-N10-18, CHN-N1-56, CHN-N2-5, CHN-L7-40, and CHN-Q5-1, respectively. The differential intracellular protein spots marked with red numbers were characterized by the LC-MS/MS analysis. pI, isoelectric point.

The 2D-GE and LC-MS/MS techniques were also used to analyze proteomics of the *V. parahaemolyticus* isolates recovered from the 12 species of aquatic products, and the obtained proteomic profiles are presented in Figure 3. The patterns yielded from three independent 2D-GE gels of each isolate were consistent (figures not shown). This analysis also revealed distinct proteomic profiles showing various visible differential protein spots (343–312) among the 12 *V. parahaemolyticus* isolates (Figures 3A–L). The *V. parahaemolyticus* CHN-N4-18 isolate recovered from *P. viridis* appeared to express the highest number of intracellular proteins (343) among all the isolates (Figure 3F). In contrast, approximately 312 intracellular proteins were observed from the proteomic profile of the CHN-B8-26 isolate from *S. strictus* (Figure 3D). Notably, for the crustaceans origin, the CHN-N1-56 isolate from *L. vannamei* expressed much more intracellular proteins (342, Figure 3I) than the CHN-N2-5 isolate from *O. oratoria* (314, Figure 3J).

### Identification of Differential Intracellular Proteins of the *V. parahaemolyticus* Isolates

Differential intracellular proteins of the 12 *V. parahaemolyticus* isolates were identified by LC-MS/MS analysis and summarized in Table 4. A total of 97 protein sequences were obtained and classified into three major GO categories in the GO database, including the biological process, cellular component, and molecular function (Supplementary Figure S2). Given that multiple biological functions could be assigned for single identified protein, the most abundant GO term was the catalytic activity (72.2%, 70/97), followed by the binding (58.8%, 57/97), cellular process (46.4%, 45/97), and metabolic process (46.4%, 45/97). The opposite patterns were cellular component organization or biogenesis (2.1%, 2/97), signaling (2.1%, 2/97), molecular function regulator (2.1%, 2/97), electron carrier activity (2.1%, 2/97), antioxidant activity (1.0%, 1/97), and multiorganism process (1.0%, 1/97) (Supplementary Figure S2A).

**TABLE 4:** Identification of the differential protein spots on the proteomic profiles of the 12 *V. parahaemolyticus* isolates by LC-MS/MS analysis.

Protein spot no.	Uniprot no.	Protein	Gene	MW (Da)	pI	Sequence coverage (%)	Putative function
P1	Q87MW0	Aldehyde-alcohol dehydrogenase	*VP2121*	97,121.96	5.73	26.78	Acetaldehyde dehydrogenase (acetylating) activity, alcohol dehydrogenase (NAD+) activity, metal ion binding, alcohol metabolic process, carbon utilization
P2	S5J2K3	Peptide ABC TSBP	*M634_12650*	63,520.87	5.35	3.70	Transmembrane transport, ATP-binding cassette (ABC) transporter complex
P3	A0A249W792	Urocanate hydratase	*hutU*	61,866.37	5.36	16.28	Urocanate hydratase activity, histidine catabolic process to glutamate and formamide, cytoplasm
P4	S5J1K8	Acetylornithine aminotransferase	*argD*	43,361.13	5.54	22.08	N2-acetyl-L-ornithine:2-oxoglutarate 5-aminotransferase activity, pyridoxal phosphate binding, arginine biosynthetic process, cytoplasm
P5	A0A0D1EDK5	Maltose/maltodextrin import ATP-binding protein MalK	*malK*	41,156.69	5.54	7.53	ATPase activity, ATPase-coupled maltose transmembrane transporter activity, ATP binding, cell membrane
P6	S5IWV0	3-Ketoacyl-CoA thiolase	*M634_20530*	41,688.1	5.2	33.25	Transferase activity, transferring acyl groups other than amino-acyl groups
P7	A0A4Q9K4T4	Aspartate-semialdehyde dehydrogenase	*asd*	40,284.89	5.2	29.11	Aspartate-semialdehyde dehydrogenase activity, NADP binding, *de novo* L-methionine biosynthetic process, diaminopimelate biosynthetic process, lysine biosynthetic process via diaminopimelate, threonine biosynthetic process
P8	A0A249W9I8	Fructose-1,6-bisphosphatase	*glpX*	35,981.09	4.95	12.84	Fructose 1,6-bisphosphate 1-phosphatase activity, metal ion binding, gluconeogenesis, glycerol metabolic process
P9	Q87L48	Putative malate oxidoreductase	*VP2767*	46,163.47	4.92	26.82	Malate dehydrogenase (decarboxylating) (NAD+) activity, metal ion binding, NAD binding
P10	A6B3V5	Phosphopentomutase	*deoB*	44,096.96	4.85	25.62	Magnesium ion binding, manganese ion binding, phosphopentomutase activity, 5-phosphoribose 1-diphosphate biosynthetic process, cellular metabolic compound salvage, deoxyribonucleotide catabolic process, cytoplasm
P11	S5J1H6	Elongation factor Tu	*tuf*	43,151.54	4.8	45.43	GTPase activity, GTP binding, translation elongation factor activity, cytoplasm
P12	A0A242UZI1	Glycerol dehydrogenase	*gldA*	38,468.69	5.05	30.56	Metal ion binding, xidoreductase activity, acting on CH-OH group of donors
P13	A0A0M9C662	Delta-aminolevulinic acid dehydratase	*ACX03_14425*	39,275.34	5.07	16.43	Metal ion binding, porphobilinogen synthase activity, porphyrin-containing compound biosynthetic process
P14	A0A0D1DR13	Membrane protein	*H323_04870*	35,567.09	4.42	19.38	Integral component of membrane, cell outer membrane
P15	A0A4S3T4K4	OmpA family protein	*E4P16_23225*	34,100.43	4.48	21.63	Membrane
P16	S5ITG7	Phosphofructokinaseompa	*fruK*	34,822.23	4.99	10.80	1-phosphofructokinase activity, ATP binding
P17	A0A4V2JS07	Succinylglutamate desuccinylase	*D5E78_01560*	38,764.47	5.39	16.37	Hydrolase activity, acting on ester bonds, succinylglutamate desuccinylase activity, zinc ion binding, arginine catabolic process to glutamate, arginine catabolic process to succinate
P18	Q87J46	Dihydroorotase	*pyrC*	37,828.45	5.56	2.63	Dihydroorotase activity, zinc ion binding, pyrimidine nucleobase biosynthetic process, *de novo* UMP biosynthetic process
P19	A0A2R9VMU2	Iron–sulfur cluster carrier protein	*C1S91_13180*	38,959.17	5.76	3.07	ATPase activity, ATP binding, iron–sulfur cluster binding, metal ion binding
P20	Q87MD9	Elongation factor Ts	*tsf*	29,773.89	5.18	14.23	Translation elongation factor activity, cytoplasm
P21	A0A0D1E4M2	5′-Nucleotidase SurE	*surE*	28,160.44	5.15	20.54	5′-nucleotidase activity, metal ion binding, nucleotide binding, cytoplasm
P22	S5J245	FMN reductase	*fre*	26,423.66	5.09	8.86	Aquacobalamin reductase activity
P23	Q87S44	Antioxidant, AhpC/Tsa family	*VP0580*	22,236.94	5.03	18.72	Peroxiredoxin activity, cell redox homeostasis, cell
P24	S5J412	Endoribonuclease L-PSP	*M634_15815*	13,800.57	4.83	33.33	—*
P25	A6B8W0	Autonomous glycyl radical cofactor	*grcA*	13,928.45	4.71	37.60	Catalytic activity
P26	A0A075BND0	Membrane protein	*ompW*	23,224.04	4.85	17.76	Outer membrane
P27	Z2E3N3	Peptidyl-prolyl *cis-trans* isomerase	*fkpA*	28,267.52	4.74	33.08	Peptidyl-prolyl *cis-trans* isomerase activity, protein folding
P28	A6B9Y8	Triosephosphate isomerase	*tpiA*	26,990.12	4.68	10.16	Triose-phosphate isomerase activity, gluconeogenesis, glycolytic process, cytoplasm
P29	A0A2R9VI74	Ribokinase	*rbsK*	32,241.19	4.58	17.38	ATP binding, metal ion binding, ribokinase activity, D-ribose catabolic process, cytoplasm
P30	Q87MS3	Putative glucose-6-phosphate 1-epimerase	*VP2158*	32,318.19	4.65	12.59	Carbohydrate binding, glucose-6-phosphate 1-epimerase activity, carbohydrate metabolic process
P31	A0A0N0CCK1	Glucose-6-phosphate isomerase	*pgi*	60,971.03	5.25	12.00	Glucose-6-phosphate isomerase activity, gluconeogenesis, glycolytic process, cytoplasm
P32	A0A0D1F327	Glycerol-3-phosphate dehydrogenase	*glpD*	59,074.41	6.1	3.24	Sn-glycerol-3-phosphate:ubiquinone-8 oxidoreductase activity, glycerol-3-phosphate metabolic process, glycerol-3-phosphate dehydrogenase complex
P33	Z2EV38	Periplasmic serine endoprotease DegP-like	*D046_1401*	43,894.46	6.2	22.28	Serine-type endopeptidase activity, periplasm
P34	Q87H06	GMP reductase	*guaC*	37,288.16	6.16	15.80	GMP reductase activity, metal ion binding, purine nucleotide metabolic process, GMP reductase complex
P35	A6BA54	Tyrosine–tRNA ligase	*tyrS*	44,069.21	5.44	11.39	ATP binding, RNA binding, tyrosine-tRNA ligase activity, tyrosyl-tRNA aminoacylation, cytoplasm
P36	Q87RK0	Phosphoenolpyruvate-protein phosphotransferase	*VP0794*	63,191.46	4.65	16.03	Kinase activity, metal ion binding, phosphoenolpyruvate-protein phosphotransferase activity, phosphoenolpyruvate-dependent sugar phosphotransferase system, cytoplasm
P37	A0A0M9C8I8	Fructose-bisphosphate aldolase	*ACX03_06815*	38,717.1	4.75	25.98	Fructose–bisphosphate aldolase activity, zinc ion binding, glycolytic process
P38	Q87MI6	Succinyl-diaminopimelate desuccinylase	*dapE*	41,037.76	4.75	4.76	Cobalt ion binding, metallopeptidase activity, succinyl-diaminopimelate desuccinylase activity, zinc ion binding, diaminopimelate biosynthetic process, lysine biosynthetic process via diaminopimelate
P39	A0A072KPG4	Succinate–CoA ligase [ADP-forming] subunit β	*sucC*	41,569.02	4.9	12.37	ATP binding, magnesium ion binding, succinate-CoA ligase (ADP-forming) activity, tricarboxylic acid cycle
P40	A0A072HGI4	Glyceraldehyde-3-phosphate dehydrogenase	*gap*	35,225.62	5.26	22.36	NAD binding, NADP binding, oxidoreductase activity, acting on the aldehyde or oxo group of donors, NAD or NADP as acceptor, glucose metabolic process
P41	A0A0L8T664	YicC family protein	*C1S91_15255*	33,216.67	5.31	12.15	—*
P42	A0A0D1FV70	Oxidoreductase	*H320_23485*	33,964.2	5.43	25.17	—*
P43	A0A0L8TW88	Threonine aldolase	*WR32_14980*	36,107.79	5.33	11.08	Lyase activity, cellular amino acid metabolic process
P44	A0A072K606	DNA-binding response regulator	*arcA*	27,034.39	5.4	12.61	DNA binding, phosphorelay signal transduction system, regulation of transcription, DNA-templated
P45	A0A0D1FP64	OmpR protein	*ompR*	27,362.26	5.79	7.95	DNA binding, phosphorelay signal transduction system, regulation of transcription, DNA-templated
P46	A6B711	Cytochrome *c* oxidase, Cbb3-type, subunit II	*ccoO*	23,607.48	5.41	14.56	Cytochrome-c oxidase activity, heme binding, metal ion binding, integral component of membrane
P47	A0A2S1MIM9	Azurin	*azu*	15,858.76	5.21	24.00	Copper ion binding, electron transfer activity, periplasmic space
P48	Q87RS3	Lipoprotein	*VP0704*	29,069.66	4.83	11.90	—*
P49	Z2EMT8	Proline dehydrogenase domain protein	*putA*	25,474.35	4.91	5.11	Oxidoreductase activity
P50	A0A0D1GLI3	Cytidylate kinase	*cmk*	24,486.65	4.94	30.97	ATP binding, cytidylate kinase activity, pyrimidine nucleotide metabolic process, cytoplasm
P51	Q87LS4	*S*-ribosylhomocysteine lyase	*luxS*	19,033.54	4.97	37.21	Iron ion binding, *S*-ribosylhomocysteine lyase activity, quorum sensing
P52	A0A242V2G1	tRNA-binding protein	*BA740_07020*	12,307.12	4.89	25.45	tRNA binding
P53	Q87Q72	Putative SpoOM-related protein	*VP1278*	27,288.79	4.88	17.41	—*
P54	A6B9V7	ATP-dependent metallopeptidase HflB	*hflB*	28,787.85	4.86	5.86	ATP binding, metalloendopeptidase activity
P55	A0A0L7YPB2	Dihydrodipicolinate synthase	*ACS91_24330*	33,972.53	4.73	5.26	Lyase activity
P56	A0A0D1EQH0	Diaminopimelate decarboxylase	*lysA*	45,889.47	5.01	7.91	Diaminopimelate decarboxylase activity, pyridoxal phosphate binding, lysine biosynthetic process via diaminopimelate
P57	A0A0F2ICT7	Cysteine desulfurase IscS	*iscS*	44,994.75	5.59	18.81	2 iron, 2 sulfur cluster binding, cysteine desulfurase activity, metal ion binding, pyridoxal phosphate binding, [2Fe–2S] cluster assembly, cytoplasm
P58	S5IT22	Aminotransferase	*M634_11710*	43,311.68	5.1	20.20	Pyridoxal phosphate binding, transaminase activity, biosynthetic process, cellular amino acid metabolic process
P59	A0A4S3T6U8	Phosphoenolpyruvate carboxylase	*E4P16_18525*	99,302.21	5.42	14.03	Magnesium ion binding, phosphoenolpyruvate carboxylase activity, carbon fixation, oxaloacetate metabolic process
P60	A0A072JVB5	Chaperone protein ClpB	*clpB*	95,871.32	5.31	32.44	ATP binding, protein metabolic process, protein refolding, response to heat, cytoplasm
P61	A0A249W7T3	Formate acetyltransferase	*pflB*	84,535.06	5.25	19.26	Formate *C*-acetyltransferase activity, carbohydrate metabolic process, cytoplasm
P62	S5JB57	4-Alpha-glucanotransferase	*M634_17555*	81,840.77	5.33	12.40	4-Alpha-glucanotransferase activity, β-maltose 4-alpha-glucanotransferase activity
P63	A0A0D1EM30	*S*-adenosylmethionine synthase	*metK*	41,990.15	5	20.57	ATP binding, magnesium ion binding, methionine adenosyltransferase activity, one-carbon metabolic process, *S*-adenosylmethionine biosynthetic process, cytoplasm
P64	A6B6D3	D-Erythrose-4-phosphate dehydrogenase	*epd*	38,248.9	5.91	8.41	Erythrose-4-phosphate dehydrogenase activity, NAD binding, pyridoxal phosphate biosynthetic process, cytoplasm
P65	A0A0D1GED2	Fructose-bisphosphate aldolase	*H334_10810*	38,880.23	4.7	32.96	Fructose-bisphosphate aldolase activity, zinc ion binding, glycolytic process
P66	A6B2R4	Thiamin pyrophospate-binding protein	*thiB*	36,582.97	4.88	5.45	Thiamine binding, thiamine transport, outer membrane-bounded periplasmic space
P67	A0A072I475	3-Oxoacyl-ACP synthase	*ACS91_26270*	42,617.75	4.95	14.14	3-Oxoacyl-[acyl-carrier-protein] synthase activity
P68	A0A0D1D1P9	DNA-directed RNA polymerase subunit alpha	*rpoA*	36,472.05	4.78	5.15	DNA binding, DNA-directed 5′-3′ RNA polymerase activity, protein dimerization activity, transcription, DNA-templated, DNA-directed RNA polymerase
P69	Z2ESY3	Outer membrane β-barrel domain protein	*D046_2887*	25,508.96	4.51	10.00	Cell outer membrane, integral component of membrane
P70	A0A0D1GU29	OmpW	*H334_14550*	23,240.1	4.85	17.76	Outer membrane
P71	Q87SD2	Putative 4-hydroxy-4-methyl-2-oxoglutarate aldolase	*VP0492*	17,708.02	4.96	21.88	4-Hydroxy-4-methyl-2-oxoglutarate aldolase activity, metal ion binding, oxaloacetate decarboxylase activity, ribonuclease inhibitor activity, regulation of RNA metabolic process
P72	A0A0L7ZQQ3	Chaperone protein DnaK	*dnaK*	69,064.13	4.69	22.45	ATP binding, unfolded protein binding, protein folding
P73	A0A0L7VQ51	Uncharacterized protein	*BA740_07860*	62,820.77	4.61	14.61	—*
P74	A0A0M3ECS2	Glyceraldehyde-3-phosphate dehydrogenase	*AAY51_01480*	35,526.09	7.01	10.88	NAD binding, NADP binding, oxidoreductase activity, acting on the aldehyde or oxo group of donors, NAD or NADP as acceptor, glucose metabolic process
P75	Q87KA1	ParB family protein	*VP3077*	32,304.73	6.49	6.14	DNA binding
P76	S5IZ22	Membrane protein	*M634_20630*	36,013.43	4.28	17.02	Cell outer membrane, integral component of membrane,
P77	A0A0D1GIY4	Porin	*H334_23665*	35,968.15	4.59	9.37	Porin activity, ion transmembrane transport, cell outer membrane
P78	A0A3E1IHF1	OmpA-like domain-containing protein	*BS585_10140*	35,983.41	4.28	22.49	Cell outer membrane, integral component of membrane,
P79	Z2ETQ8	Carbamoyl-phosphate synthase small chain	*carA*	23,039.6	4.81	15.64	Carbamoyl-phosphate synthase (glutamine-hydrolyzing) activity
P80	A6AZG0	Outer membrane protein K	*A79_4270*	30,299.56	5.11	3.31	Cell outer membrane
P81	S5IU05	Uracil phosphoribosyltransferase	*upp*	22,640.07	5.13	33.65	GTP binding, magnesium ion binding, magnesium ion binding, nucleoside metabolic process, UMP salvage, uracil salvage
P82	A0A0D1F5C1	Ribosome-recycling factor	*frr*	20,602.51	6.04	37.84	Translational termination, cytoplasm
P83	A6BCF0	Succinate dehydrogenase flavoprotein subunit	*A79_2135*	22,392.42	6.08	13.27	Succinate dehydrogenase activity
P84	Q87FQ1	Uncharacterized protein	*VPA1627*	16,587.02	6.51	11.11	—*
P85	A0A0D1E294	3-Hydroxydecanoyl-[acyl-carrier-protein] dehydratase	*fabA*	18,996.89	6.11	4.65	(3R)-hydroxymyristoyl-[acyl-carrier-protein] dehydratase activity,3-hydroxydecanoyl-[acyl-carrier-protein] dehydratase activity, trans-2-decenoyl-acyl-carrier-protein isomerase activity, fatty acid biosynthetic process, cytoplasm
P86	Q87G18	L-Lactate dehydrogenase	*lldD*	41,399.35	6.04	9.50	FMN binding, lactate dehydrogenase activity, lactate oxidation, cell inner membrane, Peripheral membrane protein, plasma membrane
P87	A0A0D1GKR8	Glycerol-3-phosphate dehydrogenase	*glpD*	58,535.82	6.09	18.88	Sn-glycerol-3-phosphate:ubiquinone-8 oxidoreductase activity, glycerol-3-phosphate metabolic process, glycerol-3-phosphate dehydrogenase complex
P88	S5J0E5	Alcohol dehydrogenase	*M634_23710*	40,190.77	5.06	24.87	Metal ion binding, oxidoreductase activity
P89	A0A0D1F963	Pyruvate dehydrogenase E1 component subunit alpha	*pdhA*	40,236.89	5.1	12.64	Pyruvate dehydrogenase (acetyl-transferring) activity, glycolytic process
P90	A6B1F6	Inorganic diphosphatase	*A79_0160*	33,028.53	4.59	13.95	Metal ion binding, pyrophosphatase activity, cytoplasm
P91	S5IS60	Acetyltransferase	*M634_19235*	20,444.06	5.27	28.26	Acetyltransferase activity
P92	Z2EFM2	Single-stranded DNA-binding protein	*ssb*	19,563.39	5.18	31.25	Single-stranded DNA binding, DNA recombination, DNA repair, DNA replication
P93	A0A2S1MAR5	tRNA-specific 2-thiouridylase MnmA	*mnmA*	42,212.27	5.11	14.17	ATP binding, methyltransferase activity, sulfur transferase activity, tRNA binding, tRNA modification, cytoplasm
P94	A6B5M0	*S*-(hydroxymethyl) glutathione dehydrogenase	*A79_5858*	40,775.48	5.17	23.82	*S*-(hydroxymethyl)glutathione dehydrogenase activity, zinc ion binding, ethanol oxidation
P95	S5IUX8	Phosphoglycerate kinase	*pgk*	40,732.24	4.9	15.28	ATP binding, phosphoglycerate kinase activity, glycolytic process, cytoplasm
P96	A0A242V2H0	2,3,4,5-Tetrahydropyridine-2,6-dicarboxylate *N*-succinyltransferase	*dapD*	35,626	4.99	17.20	2,3,4,5-Tetrahydropyridine-2,6-dicarboxylate *N*-succinyltransferase activity, magnesium ion binding, diaminopimelate biosynthetic process, lysine biosynthetic process via diaminopimelate, cytoplasm
P97	A0A072L0V1	Transaldolase	*tal*	34,805.45	4.86	37.03	Sedoheptulose-7-phosphate: D-glyceraldehyde-3-phosphate glyceronetransferase activity, carbohydrate metabolic process, pentose-phosphate shunt, cytoplasm

*—, not detected.

### Proteomic Comparison of the *V. parahaemolyticus* Isolates From Three Kinds of Aquatic Products

The proteomic profiles of the shellfish, crustaceans, and fish origins were also different (Figure 3). Interestingly, approximately 28.9% (28/97) of the differential Intracellular proteins only appeared on the proteomic profiles of the *V. parahaemolyticus* isolates from the eight species of shellfish, but absent from the crustaceans and fish. Of these proteins, the glycerol dehydrogenase (GldA, Spot P12), 5′-nucleotidase surE (Spot P21), and putative glucose-6-phosphate 1-epimerase (G6PE, Spot P30) were shown only on the proteomic profile of the CHN-B2-28 isolate from *R. philippinarum*. The GldA (Spot P12) is required to catalyze the first step in fermentative glycerol metabolism, and its product is then funneled into the glycolytic pathway for further degradation. The 5′-nucleotidase surE (Spot P21) has nucleotidase activity and is involved in nucleotide metabolism in *Escherichia coli* (Proudfoot et al., 2004). The G6PE (Spot P30) catalyzes glucose-6-phosphate to fructose-6-phosphate that converted to fructose-1,6-bisphosphate under the catalysis of PFK (Spot P16). In this study, seven intracellular proteins were found only on the proteomic profile of the CHN-B5-29 isolate from *P. magellanicus*, including the periplasmic serine endoprotease DegP-like (Spot P33), GAPDH (Spot P40), oxidoreductase (Spot P42), threonine aldolase (Spot P43), azurin (Spot P47), *s*-ribosylhomocysteine lyase (LuxS, Spot P51), and tRNA-binding protein (Spot P52). Among these proteins, for instance, the oxidoreductase (Spot P42) facilitates the detoxification of xenobiotic organic compounds by various microorganisms (Khatoon et al., 2017). The threonine aldolase (Spot P43) catalyzes the cleavage of threonine into glycine and acetaldehyde and involves in threonine decomposition and glycine synthesis (Liu et al., 2015). Azurin (Spot P47) is necessary for bacterial protection from oxidative stress (electron donor to nitrate reductase) and copper toxicity in *P. aeruginosa* (Mohammadi-Barzelighi et al., 2019). The LuxS (Spot P51) is highly conserved among *Aeromonas hydrophila*, *E. coli*, *V. cholerae*, and *Vibrio harveyi* and has a critical role in regulation of genes associated with the transport of nucleotides, metabolism, and synthesis of cell walls or membranes. In this study, the cysteine desulfurase IscS (Spot P57) was observed only on the proteomic profile of the CHN-B6-62 isolate from *S. constricta*, which was involved in iron–sulfur cluster biogenesis and oxidative stress defense in *Mycobacterium tuberculosis* (Rybniker et al., 2014). Additionally, the CHN-N4-18 isolate from *P. viridis* expressed the outer membrane β-barrel domain protein (Spot P69), OmpW (Spot P70), and putative 4-hydroxy-4-methyl-2-oxoglutarate aldolase (Spot P71) (Table 4).

For the *V. parahaemolyticus* isolates of the crustaceans origin, six differential intracellular proteins appeared only on the proteomic profile of the CHN-N1-56 isolate from *L. vannamei*, including the ParB family protein (Spot P75), porin (Spot P77), outer membrane protein A (OmpA)–like domain-containing protein (Spot P78), uracil phosphoribosyltransferase (UPRT, Spot P81), ribosome-recycling factor (Spot P82), and an uncharacterized protein (Spot P84) encoded by the *VPA1627* gene of *V. parahaemolyticus* RIMD 2210633 strain (Table 4). Recent research has indicated that the UPRT (Spot P81) converts uracil to uridine monophosphate in the pyrimidine salvage pathway in the presence of phosphoribosyl pyrophosphate (Silva et al., 2019). Additionally, the carbamoyl-phosphate synthase small chain (Spot 79) was only expressed by the CHN-N1-56 and CHN-N2-5 isolates from *L. vannamei* and *O. oratoria*, respectively (Table 4).

For the *V. parahaemolyticus* isolates of the fish origin, six differential intracellular proteins were expressed only by the CHN-Q5-1 isolate from *C. idellus*, including the OmpK (Spot P80), alcohol dehydrogenase (Spot P88), pyruvate dehydrogenase E1 component subunit alpha (Spot P89), inorganic diphosphatase (Spot P90), acetyltransferase (Spot P91), and single-stranded DNA-binding protein (SSB, Spot P92). The acetyltransferase (Spot P91) acts on acetylation of amino acids, which determines vital regulatory processes (Christensen et al., 2019). In addition, three intracellular proteins were only expressed by the CHN-L7-40 isolate from *A. nobilis*, including the tRNA-specific 2-thiouridylase MnmA (Spot P93), *S*-(hydroxymethyl) glutathione dehydrogenase (Spot P94), 2,3,4,5-tetrahydropyridine-2,6-dicarboxylate *N*-succinyltransferase (Spot P96), and transaldolase (Spot P97) (Table 4).

### Proteomic Comparison of the *V. parahaemolyticus* Isolates With Pathogenic Reference Strains

The proteomic profiles of the *V. parahaemolyticus* ATCC33847 and ATCC17802 strains were also obtained (Supplementary Figure S3). These toxic strains of clinical origin appeared to express more intracellular proteins (459–462) than the 12 *V. parahaemolyticus* isolates of aquatic product origins (312–343). Comparative proteomic analysis revealed that approximately 23.7% (23/97) of the differential intracellular proteins expressed by the 12 *V. parahaemolyticus* isolates (Table 4) were observed at similar locations on the proteomic profiles of the ATCC33847 and the ATCC17802 strains (Supplementary Figure S3), including three membrane-related proteins: OmpA family protein (Spot P15), cytochrome c oxidase (Spot P46), and lipoprotein (Spot P48); 10 metabolism-related proteins: aldehyde-alcohol dehydrogenase (AdhE) (Spot P1), PFK (Spot P16), succinylglutamate desuccinylase (Spot P17), FMN reductase (Spot P22), autonomous glycyl radical cofactor (Spot P25), triosephosphate isomerase (Spot P28), glucose-6-phosphate isomerase (Spot P31), glycerol-3-phosphate dehydrogenase (Spot P32), GAPDH (Spot P40), and cytidylate kinase (Spot P50); three biosynthesis-related proteins: ASD (Spot P7), endoribonuclease L-PSP (Spot P24), and tRNA-binding protein (Spot P52); two translation-associated proteins: EF-Tu (Spot P11) and an EF-Ts (Spot P20); one transport protein [phosphoenolpyruvate-protein phosphotransferase (PtsA), Spot P36]; and four bacterial cell protection factors: antioxidant, alkyl hydroperoxide reductase C (AhpC)/Tsa family (AhpC) (Spot P23), periplasmic serine endoprotease DegP-like (Spot P33), cytidylate kinase (Spot P50), and ATP-dependent metallopeptidase HflB (Spot P54) (Table 4).

Notably, among the differential intracellular proteins identified in this study, some were reported to be involved in the virulence of pathogenic bacteria, including the AdhE (Spot P1), OmpAs (Spots P15 and P78), AhpC (Spot P23), PtsA (Spot P36), and phosphoglycerate kinase (PGK) (Spot P95). The latter catalyzes the transfer of a phosphate group from 1,3-diphosphoglycerate to ADP to produce 3-phosphoglycerate and ATP (Smith et al., 2011).

### Effects of Aquatic Product Matrix on Proteomic Profiles of the *V. parahaemolyticus* Isolates

**FIGURE 4:**
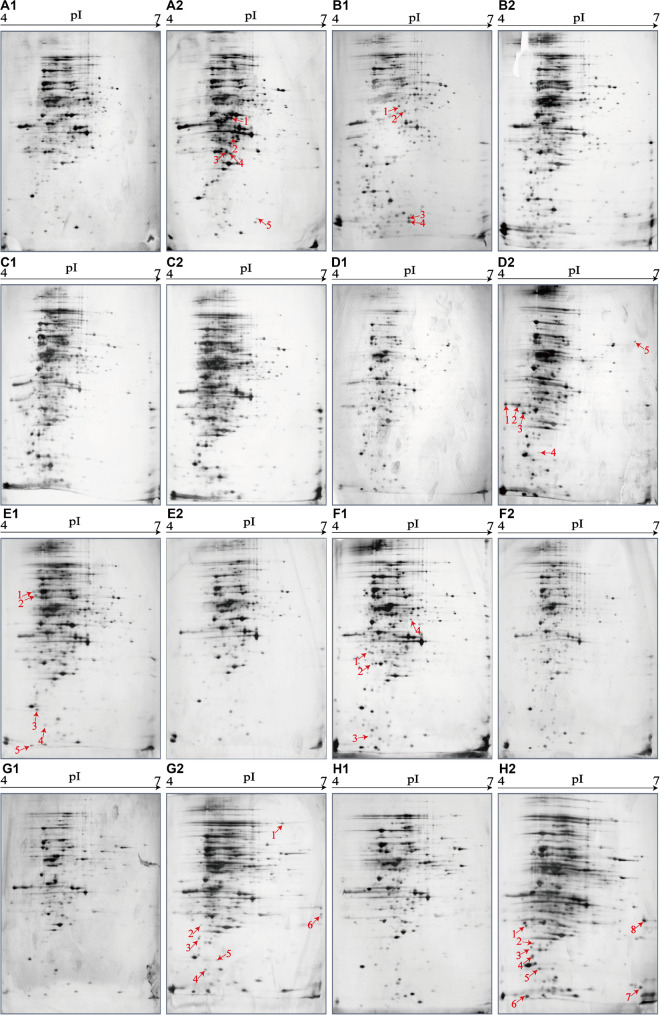
Effects of aquatic product matrices on proteomic profiles of the *V. parahaemolyticus* isolates of the shellfish origin. **(A1–H1)** *V. parahaemolyticus* CHN-2-28, CHN-B5-29, CHN-B6-62, CHN-B8-26, CHN-N3-2, CHN-N4-18, CHN-N8-5, and CHN-N10-18 isolates incubated in the TSB medium (pH 8.5, 3% NaCl) at 37°C, respectively. **(A2–H2)** The *V. parahaemolyticus* isolates were incubated in the *R. philippinarum*, *P. magellanicus*, *S. constricta*, *S. strictus*, *P. undulate*, *P. viridis*, *M. veneriformis*, and *O. gigas thunberg* matrices media at 37°C, respectively.

**FIGURE 5:**
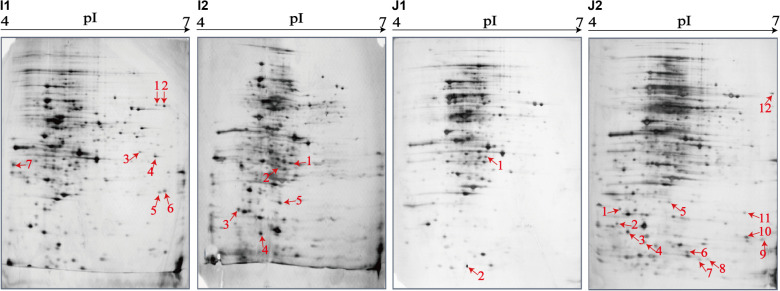
Effects of aquatic product matrix on proteomic profiles of the *V. parahaemolyticus* isolates of the crustaceans origin. **(I_1_,J_1_)** *V. parahaemolyticus* CHN-N1-56 and N2-5 isolates incubated in the TSB medium (pH 8.5, 3% NaCl) at 37°C, respectively. **(I_2_,J_2_)** the CHN-N1-56 and N2-5 isolates incubated in the *L. vannamei* and *O. oratoria* matrices media at 37°C, respectively.

**FIGURE 6:**
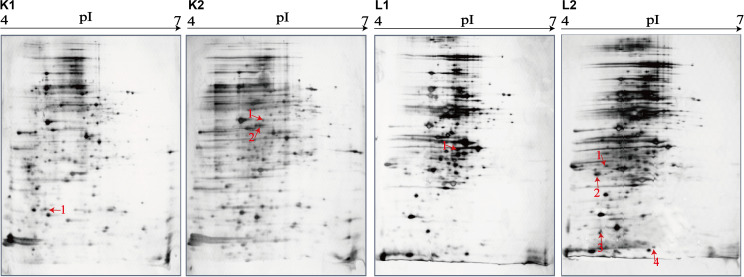
Effects of aquatic product matrix on proteomic profiles of the *V. parahaemolyticus* isolates of the fish origin. **(K_1_,L_1_)** *V. parahaemolyticus* CHN-L7-40 and Q5-1 isolates incubated in the TSB medium (pH 8.5, 3% NaCl) at 37°C, respectively. **(K_2_,L_2_)** the CHN-L7-40 and Q5-1 isolates incubated in the *A. nobilis* and *C. idellus* matrices media at 37°C, respectively.

To get insights into implications of aquatic product matrices on the proteomes of the 12 *V. parahaemolyticus* isolates, we incubated these isolates in their corresponding matrix media and identified a total of 71 differential intracellular proteins by the 2D-GE and LC-MS/MS analysis (Figures 4–6 and Table 5). These proteins were classified into three major GO categories, in which the most abundant GO term was catalytic activity (62.0%, 44/71), followed by cell (50.7%, 36/71) and binding (49.3%, 35/71). The opposite patterns were biological regulation (1.4%, 1/71), organelle (1.4%, 1/71), and structural molecule activity (1.4%, 1/71) (Supplementary Figure S2B).

**TABLE 5:** Identification of the differential proteins of the 12 *V. parahaemolyticus* isolates incubated between in the TSB and aquatic product matrix media.

Protein spot no.	Uniprot no.	Protein	Gene	MW (Da)	pI	Sequence coverage (%)	Putative function	*V. parahaemolyticus* isolate/matrix medium
A2-1	A0A242UZI1	Glycerol dehydrogenase	*gldA*	38,468.69	5.05	30.56	Metal ion binding, xidoreductase activity, acting on CH-OH group of donors	CHN-B2-28/*R. philippinarum*
A2-2	A0A249W9I8	Fructose-1,6-bisphosphatase	*glpX*	35,981.09	4.95	12.84	Fructose 1,6-bisphosphate 1-phosphatase activity, metal ion binding, gluconeogenesis, glycerol metabolic process	CHN-B2-28/*R. philippinarum*
A2-3	Q87M78	2,3,4,5-tetrahydropyridine-2,6-dicarboxylate *N*-succinyltransferase	*dapD*	35,639.98	4.99	7.00	2,3,4,5-Tetrahydropyridine-2,6-dicarboxylate *N*-succinyltransferase activity, magnesium ion binding, diaminopimelate biosynthetic process, lysine biosynthetic process via diaminopimelate, cytoplasm	CHN-B2-28/*R. philippinarum*
A2-4	A0A0D1EXH8	Choline ABC TSBP	*H334_11605*	34,297.4	5.06	15.71	Choline binding, transmembrane transporter activity, choline transport, ATP-binding cassette (ABC) transporter complex, periplasmic space	CHN-B2-28/*R. philippinarum*
A2-5	A6B711	Cytochrome *c* oxidase, Cbb3-type, subunit II	*ccoO*	23,607.48	5.41	14.56	Cytochrome-*c* oxidase activity, heme binding, metal ion binding, integral component of membrane	CHN-B2-28/*R. philippinarum*
B1-1	Q87MN7	3-oxoacyl-[acyl-carrier-protein] synthase I	*VP2194*	42,617.75	4.95	30.02	Transferase activity	CHN-B5-29/TSB medium
B1-2	A0A0D1EM30	*S*-adenosylmethionine synthase	*metK*	41,990.15	5	20.57	ATP binding, magnesium ion binding, methionine adenosyltransferase activity, one-carbon metabolic process, *S*-adenosylmethionine biosynthetic process, cytoplasm	CHN-B5-29/TSB medium
B1-3	A0A2S1MIM9	Azurin	*azu*	15,858.76	5.21	24.00	Copper ion binding, electron transfer activity, periplasmic space	CHN-B5-29/TSB medium
B1-4	S5IID9	Universal stress protein	*M634_02380*	15,681.81	5.19	29.79	Cytoplasm	CHN-B5-29/TSB medium
D2-1	S5IZ22	Outer membrane protein A (OmpA) family	*M634_20630*	36,013.43	4.28	17.02	Cell outer membrane, integral component of membrane	CHN-B8-26/*S. strictus*
D2-2	A0A072JT35	OmpA family protein	*ACS91_20150*	35,567.09	4.42	7.08	Cell outer membrane, integral component of membrane	CHN-B8-26/*S. strictus*
D2-3	A0A4S3T4K4	OmpA family protein	*E4P16_23225*	34,100.43	4.48	21.63	Membrane	CHN-B8-26/*S. strictus*
D2-4	A6B9V7	ATP-dependent metallopeptidase HflB	*hflB*	28,787.85	4.86	5.86	ATP binding, metalloendopeptidase activity	CHN-B8-26/*S. strictus*
D2-5	A0A0D1F327	Glycerol-3-phosphate dehydrogenase	*glpD*	59,074.41	6.1	3.24	Sn-glycerol-3-phosphate:ubiquinone-8 oxidoreductase activity, glycerol-3-phosphate metabolic process, glycerol-3-phosphate dehydrogenase complex	CHN-B8-26/*S. strictus*
E1-1	A0A0L7ZQQ3	Chaperone protein DnaK	*dnaK*	69,064.13	4.69	22.45	ATP binding, unfolded protein binding, protein folding	CHN-N3-2/TSB medium
E1-2	Q87RK0	Phosphoenolpyruvate-protein phosphotransferase	*VP0794*	63,191.46	4.65	16.03	Kinase activity, metal ion binding, phosphoenolpyruvate-protein phosphotransferase activity, phosphoenolpyruvate-dependent sugar phosphotransferase system, cytoplasm	CHN-N3-2/TSB medium
E1-3	A0A242UZU9	Triosephosphate isomerase	*tpiA*	26,989.18	4.78	21.88	Triose-phosphate isomerase activity, gluconeogenesis, glycolytic process, cytoplasm	CHN-N3-2/TSB medium
E1-4	A0A075BND0	Outer membrane protein W	*ompW*	23,224.04	4.85	17.76	Outer membrane	CHN-N3-2/TSB medium
E1-5	A6B8W0	Autonomous glycyl radical cofactor	*grcA*	13,928.45	4.71	37.60	Catalytic activity	CHN-N3-2/TSB medium
F1-1	Q87SZ0	DNA-directed RNA polymerase subunit alpha	*rpoA*	36,472.05	4.78	11.21	DNA binding, DNA-directed 5′-3′ RNA polymerase activity, protein dimerization activity, transcription, DNA-templated, DNA-directed RNA polymerase	CHN-N4-18/TSB medium
F1-2	Q87T56	ADP-L-glycero-D-manno-heptose-6-epimerase	*hldD*	35,215.99	4.86	6.71	ADP-glyceromanno-heptose 6-epimerase activity; NADP binding; ADP-L-glycero-β-D-manno-heptose biosynthetic process; lipopolysaccharide core region biosynthetic process	CHN-N4-18/TSB medium
F1-3	A0A0D1GU29	Outer membrane protein W	*H334_14550*	23,240.1	4.85	17.76	Outer membrane	CHN-N4-18/TSB medium
F1-4	A0A2S1MAR5	tRNA-specific 2-thiouridylase MnmA	*mnmA*	42,212.27	5.11	14.17	ATP binding, methyltransferase activity, sulfur transferase activity, tRNA binding, tRNA modification, cytoplasm	CHN-N4-18/TSB medium
G2-1	Q87MW0	Aldehyde-alcohol dehydrogenase	*VP2121*	97,121.96	5.73	26.78	Acetaldehyde dehydrogenase (acetylating) activity, alcohol dehydrogenase (NAD+) activity, metal ion binding, alcohol metabolic process, carbon utilization	CHN-N8-5/*M. veneriformis*
G2-2	A0A0N0CBA6	Uncharacterized protein	*ACX03_16865*	33,357.07	4.39	6.35	—*	CHN-N8-5/*M. veneriformis*
G2-3	A0A2R9VI74	Ribokinase	*rbsK*	32,241.19	4.58	17.38	ATP binding, metal ion binding, ribokinase activity, D-ribose catabolic process, cytoplasm	CHN-N8-5/*M. veneriformis*
G2-4	A0A075BND0	Outer membrane protein W	*ompW*	23,224.04	4.85	17.76	Outer membrane	CHN-N8-5/*M. veneriformis*
G2-5	Z2EMT8	Proline dehydrogenase domain protein	*putA*	25,474.35	4.91	5.11	Oxidoreductase activity	CHN-N8-5/*M. veneriformis*
G2-6	A0A0M3ECS2	Glyceraldehyde-3-phosphate dehydrogenase	*AAY51_01480*	35,526.09	7.01	10.88	NAD binding, NADP binding, oxidoreductase activity, acting on the aldehyde or oxo group of donors, NAD or NADP as acceptor, glucose metabolic process	CHN-N8-5/*M. veneriformis*
H2-1	A0A4S3T4K4	OmpA family protein	*E4P16_23225*	34,100.43	4.48	21.63	Membrane	CHN-N10-18/*O. gigas thunberg*
H2-2	Q87MS3	Putative glucose-6-phosphate 1-epimerase	*VP2158*	32,318.19	4.65	12.59	Carbohydrate binding, glucose-6-phosphate 1-epimerase activity, carbohydrate metabolic process	CHN-N10-18/*O. gigas thunberg*
H2-3	A0A2R9VI74	Ribokinase	*rbsK*	32,241.19	4.58	17.38	ATP binding, metal ion binding, ribokinase activity, D-ribose catabolic process, cytoplasm	CHN-N10-18/*O. gigas thunberg*
H2-4	Z2E3N3	Peptidyl-prolyl *cis*-*trans* isomerase	*fkpA*	28,267.52	4.74	33.08	Peptidyl-prolyl *cis*-*trans* isomerase activity, protein folding	CHN-N10-18/*O. gigas thunberg*
H2-5	A0A242UZU9	Triosephosphate isomerase	*tpiA*	26,989.18	4.78	21.88	Triose-phosphate isomerase activity, gluconeogenesis, glycolytic process, cytoplasm	CHN-N10-18/*O. gigas thunberg*
H2-6	A6B8W0	Autonomous glycyl radical cofactor	*grcA*	13,928.45	4.71	37.60	Catalytic activity	CHN-N10-18/*O. gigas thunberg*
H2-7	Q87FQ1	Uncharacterized protein	*VPA1627*	16,587.02	6.51	11.11	—*	CHN-N10-18/*O. gigas thunberg*
H2-8	A0A0M3ECS2	Glyceraldehyde-3-phosphate dehydrogenase	*AAY51_01480*	35,526.09	7.01	19.03	NAD binding, NADP binding, oxidoreductase activity, acting on the aldehyde or oxo group of donors, NAD or NADP as acceptor	CHN-N10-18/*O. gigas thunberg*
I1-1	A0A0D1EXL2	Inosine-5′-monophosphate dehydrogenase	*guaB*	51,685.5	6.06	38.52	IMP dehydrogenase activity, metal ion binding, nucleotide binding, GMP biosynthetic process	CHN-N1-56/TSB medium
I1-2	A0A0L7Z783	Formate transporter	*ACS91_17705*	52,146.53	6.09	6.83	Integral component of membrane, formate transmembrane transporter activity	CHN-N1-56/TSB medium
I1-3	A6B6D3	D-Erythrose-4-phosphate dehydrogenase	*epd*	38,248.9	5.91	8.41	Erythrose-4-phosphate dehydrogenase activity, NAD binding, pyridoxal phosphate biosynthetic process, cytoplasm	CHN-N1-56/TSB medium
I1-4	Q87H06	GMP reductase	*guaC*	37,288.16	6.16	15.80	GMP reductase activity, metal ion binding, purine nucleotide metabolic process, GMP reductase complex	CHN-N1-56/TSB medium
I1-5	A0A0D1DUY5	Acyl-CoA thioesterase II	*H320_00530*	32,641.74	6.42	4.90	Acyl-CoA hydrolase activity	CHN-N1-56/TSB medium
I1-6	Q87KA1	ParB family protein	*VP3077*	32,304.73	6.49	6.14	DNA binding	CHN-N1-56/TSB medium
I1-7	S5IZ22	OmpA family protein	*M634_20630*	36,013.43	4.28	17.02	Cell outer membrane, integral component of membrane,	CHN-N1-56/TSB medium
I2-1	A0A242V2H0	2,3,4,5-Tetrahydropyridine-2,6-dicarboxylate *N*-succinyltransferase	*dapD*	35,626	4.99	17.20	2,3,4,5-tetrahydropyridine-2,6-dicarboxylate *N*-succinyltransferase activity, magnesium ion binding, diaminopimelate biosynthetic process, lysine biosynthetic process via diaminopimelate, cytoplasm	CHN-N1-56/*L. vannamei*
I2-2	S5IZ25	Transaldolase	*tal*	34,805.45	4.86	10.76	Sedoheptulose-7-phosphate:D-glyceraldehyde-3-phosphate glyceronetransferase activity, carbohydrate metabolic process, pentose-phosphate shunt, cytoplasm	CHN-N1-56/*L. vannamei*
I2-3	A6B3V3	Deoxyribose-phosphate aldolase	*deoC*	27,744.45	4.68	13.18	Deoxyribose-phosphate aldolase activity, carbohydrate catabolic process, deoxyribonucleotide catabolic process, deoxyribose phosphate catabolic process, cytoplasm	CHN-N1-56/*L. vannamei*
I2-4	A0A075BND0	Out membrane protein W	*ompW*	23,224.04	4.85	17.76	Outer membrane	CHN-N1-56/*L. vannamei*
I2-5	Q87RS3	Lipoprotein	*VP0704*	29,069.66	4.83	11.90	—*	CHN-N1-56/*L. vannamei*
J1-1	A0A0D1EXH8	Glycine betaine-binding protein OpuAC	*H334_11605*	34,297.4	5.06	15.71	Choline binding, transmembrane transporter activity, choline transport, ATP-binding cassette (ABC) transporter complex, periplasmic space	CHN-N2-5/TSB medium
J1-2	A0A0D1V969	DNA polymerase sliding clamp subunit	*ACS91_16100*	18,275.43	5.07	18.24	Ferric iron binding, oxidoreductase activity, oxidizing metal ions, cellular iron ion homeostasis, cell	CHN-N2-5/TSB medium
J2-1	A6B3V3	Deoxyribose-phosphate aldolase	*deoC*	27,744.45	4.68	13.18	Deoxyribose-phosphate aldolase activity, carbohydrate catabolic process, deoxyribonucleotide catabolic process, deoxyribose phosphate catabolic process, cytoplasm	CHN-N2-5/*O. oratoria*
J2-2	Z2EUM4	Phosphoribosyltransferase	*hisG*	24,091.48	4.66	19.46	ATP phosphoribosyltransferase activity, magnesium ion binding, histidine biosynthetic process, cytoplasm	CHN-N2-5/*O. oratoria*
J2-3	Z2ESY3	Outer membrane β-barrel domain protein	*D046_2887*	25,508.96	4.51	10.00	Cell outer membrane, integral component of membrane	CHN-N2-5/*O. oratoria*
J2-4	A0A0D1EJJ6	Protein GrpE	*grpE*	22,368.9	4.54	13.64	Adenyl-nucleotide exchange factor activity, chaperone binding, protein homodimerization activity, protein folding, cytoplasm	CHN-N2-5/*O. oratoria*
J2-5	Q87RS3	Lipoprotein	*VP0704*	29,069.66	4.83	11.90	—*	CHN-N2-5/*O. oratoria*
J2-6	A0A2S1MIM9	Azurin	*azu*	15,858.76	5.21	24.00	Copper ion binding, electron transfer activity, periplasmic space	CHN-N2-5/*O. oratoria*
J2-7	Q87RU4	6,7-Dimethyl-8-ribityllumazine synthase	*ribH*	16,431.67	5.37	38.46	6,7-dimethyl-8-ribityllumazine synthase activity, riboflavin biosynthetic process	CHN-N2-5/*O. oratoria*
J2-8	A0A0D1F6F7	Histidine triad nucleotide-binding protein	*H334_15790*	12,987.93	5.45	45.69	Catalytic activity	CHN-N2-5/*O. oratoria*
J2-9	Q87FQ1	Uncharacterized protein	*VPA1627*	16,587.02	6.51	11.11	—*	CHN-N2-5/*O. oratoria*
J2-10	A0A0D1E294	3-Hydroxydecanoyl-[acyl-carrier-protein] dehydratase	*fabA*	18,996.89	6.11	4.65	(3R)-hydroxymyristoyl-[acyl-carrier-protein] dehydratase activity,3-hydroxydecanoyl-[acyl-carrier-protein] dehydratase activity, trans-2-decenoyl-acyl-carrier-protein isomerase activity, fatty acid biosynthetic process, cytoplasm	CHN-N2-5/*O. oratoria*
J2-11	A6BCF0	Succinate dehydrogenase flavoprotein subunit	*A79_2135*	22,392.42	6.08	13.27	Succinate dehydrogenase activity	CHN-N2-5/*O. oratoria*
J2-12	A0A0D1F327	Glycerol-3-phosphate dehydrogenase	*glpD*	59,074.41	6.1	3.24	Sn-glycerol-3-phosphate:ubiquinone-8 oxidoreductase activity, glycerol-3-phosphate metabolic process, glycerol-3-phosphate dehydrogenase complex	CHN-N2-5/*O. oratoria*
K1-1	A6B9V7	ATP-dependent metallopeptidase HflB	*hflB*	28,787.85	4.86	5.86	ATP binding, metalloendopeptidase activity	CHN-L7-40/TSB medium
K2-1	A0A0D1EM30	*S*-adenosylmethionine synthase	*metK*	41,990.15	5	20.57	ATP binding, magnesium ion binding, methionine adenosyltransferase activity, one-carbon metabolic process, *S*-adenosylmethionine biosynthetic process, cytoplasm	CHN-L7-40/*A. nobilis*
K2-2	Q87MI6	Succinyl-diaminopimelate desuccinylase	*dapE*	41,037.76	4.75	4.76	Cobalt ion binding, metallopeptidase activity, succinyl-diaminopimelate desuccinylase activity, zinc ion binding, diaminopimelate biosynthetic process, lysine biosynthetic process via diaminopimelate	CHN-L7-40/*A. nobilis*
L1-1	A0A0N0UKW5	Aspartate carbamoyltransferase	*pyrB*	34,363.55	5.03	6.47	Amino acid binding, aspartate carbamoyltransferase activity, *de novo* pyrimidine nucleobase biosynthetic process, *de novo* UMP biosynthetic process, cellular amino acid metabolic process	CHN-Q5-1/TSB medium
L2-1	A0A0L7YIN7	3-Chlorobenzoate-3,4-dioxygenase dehydrogenase	*WR32_06855*	38,032.68	4.62	7.96	Dioxygenase activity	CHN-Q5-1/*C. idellus*
L2-2	A0A4S3T4K4	OmpA family protein	*E4P16_23225*	34,100.43	4.48	21.63	Cell outer membrane; integral component of membrane	CHN-Q5-1/*C. idellus*
L2-3	S5IJL9	Protein GrpE	*grpE*	22,368.9	4.54	50.51	Adenyl-nucleotide exchange factor activity; chaperone binding; protein homodimerization activity; protein folding; cytoplasm	CHN-Q5-1/*C. idellus*
L2-4	A0A0D1ESD7	50S ribosomal protein L9	*rplI*	15,708.8	5.19	66.00	Ribosome; rRNA binding; structural constituent of ribosome; translation	CHN-Q5-1/*C. idellus*

*—, not detected.

The shellfish matrix media obviously changed the proteomic profiles of the 8 *V. parahaemolyticus* isolates of the shellfish origin (Figures 4A_**2**_–H_**2**_). For instance, approximately 319 and 326 intracellular protein spots were observed on the proteomic profiles of the CHN-N10-28 isolate grown in the TSB and *O. gigas thunberg* matrix media, respectively (Figures 4H_**1**_,H_**2**_). Among these, eight intracellular proteins were expressed by the CHN-N10-28 isolate grown in the *O. gigas thunberg* matrix medium (Figure 4H_**2**_), including the OmpA family protein (Spot H2-1), putative glucose-6-phosphate 1-epimerase (Spot H2-2), ribokinase (Spot H2-3), peptidyl-prolyl *cis-trans* isomerase (Spot H2-4), triosephosphate isomerase (Spot H2-5), autonomous glycyl radical cofactor (Spot H2-6), GAPDH (Spot H2-8), and an uncharacterized protein (Spot H2-7) encoded by the *VPA1627* gene of *V. parahaemolyticus* RIMD 2210633 strain with currently unknown functions in public databases (Table 5).

For the crustacean matrix media, for example, approximately 314 and 325 intracellular proteins were produced by the CHN-N2-5 isolate grown in the TSB and *O. oratoria* matrix media, respectively (Figures 5J_**1**_,J_**2**_). Two differential intracellular proteins were expressed by the CHN-N2-5 isolate grown in the former medium (Figure 5J_**1**_), including the glycine betaine-binding protein OpuAC (Spot J1-1), and DNA polymerase sliding clamp subunit (Spot J1-2). Remarkably, approximately 12 differential intracellular proteins were expressed by the CHN-N2-5 isolate grown in the *O. oratoria* matrix medium (Figure 5J_**2**_), including the deoxyribose-phosphate aldolase (Spot J2-1), phosphoribosyltransferase (Spot J2-2), outer membrane β-barrel domain protein (Spot J2-3), GrpE (Spot J2-4), lipoprotein (Spot J2-5), azurin (Spot J2-6), 6,7-dimethyl-8-ribityllumazine synthase (Spot J2-7), histidine triad nucleotide-binding protein (Spot J2-8), 3-hydroxydecanoyl-(acyl-carrier-protein) dehydratase (Spot J2-10), succinate dehydrogenase flavoprotein subunit (Spot J2-11), glycerol-3-phosphate dehydrogenase (Spot J2-12), and an uncharacterized protein (Spot J2-9) encoded by the *VPA1627* gene of *V. parahaemolyticus* RIMD 2210633 strain (Table 5).

Likewise, for the fish matrix media, for instance, approximately 329 and 333 intracellular protein spots were observed on the proteomic profiles derived from the CHN-Q5-1 isolate grown in the TSB and *C. idellus* matrix media, respectively (Figures 6L_**1**_,L_**2**_). The aspartate carbamoyltransferase (Spot L1-1) was expressed by the CHN-Q5-1 isolate grown in the former medium (Figure 6L_**1**_), whereas four were produced in the *C. idellus* matrix medium (Figure 6L_**2**_), including the 3-chlorobenzoate-3,4-dioxygenase dehydrogenase (Spot L2-1), OmpA family protein (Spot L2-2), GrpE (Spot L2-3), and 50S ribosomal protein L9 (Spot L2-4) (Table 5). Additionally, to validate the differential proteins induced by the aquatic product matrices, we examined gene expression of several representative proteins by the quantitative RT-PCR assay. The resulting data were generally consistent with the proteomic analysis (Supplementary Figure S4).

### Aquatic Product Matrix Composition

As shown in Supplementary Figure S5, the protein (3.73‰–0.43‰), carbohydrate (1.23‰–0.01‰), and crude fat (1.00‰–0.01‰) contents of the 12 types of aquatic product matrices were remarkably different. The protein concentration in the *P. undulate* matrix was the highest (3.73‰), followed by 3.44‰ in the *P. viridis*, and 3.25‰ in the *L. vannamei* matrices, whereas the opposite patterns were observed in the *P. magellanicus* (1.50‰), *S. strictus* (1.36‰), and *O. gigas thunberg* (0.43‰) matrices. The fat contents of the aquatic product matrices were much higher in the *L. vannamei* (1.01‰), *O. oratoria* (0.85‰), and *P. magellanicus* (0.84%) than those in the *P. undulate* (0.36%), *S. strictus* (0.31%), and *S. constricta* (0.01%) matrices. The carbohydrate content of the *P. viridis* (1.23‰) matrix was the highest, whereas that of the *O. gigas thunberg* (0.01‰) matrix was the lowest (Supplementary Figure S5).

## Discussion

*Vibrio parahaemolyticus* is the leading seafoodborne pathogenic bacterium worldwide. Nevertheless, the information in *V. parahaemolyticus* proteomics is minimally available to date (Fu et al., 2014; He et al., 2015; Perez-Acosta et al., 2018; Tang et al., 2018; Zhong et al., 2019). Based on our prior studies, in this study, we obtained and compared the secretomic and proteomic profiles of the *V. parahaemolyticus* isolates recovered from 12 species of commonly consumed aquatic products by 2D-GE and LC-MS/MS analysis.

Secreted proteins have a major role in the pathogenesis of bacterial infection of host cells. He et al. (2015) investigated secretomic profiles derived from *V. parahaemolyticus* strains isolated from five species of aquatic products and identified six extracellular virulence-associated proteins involved in the pathogenicity of bacteria, such as EF-Tu, pyridoxine 5′-phosphate synthase, σ54 modulation protein, dihydrolipoyl dehydrogenase, transaldolase, and PGK. Among these proteins, the EF-Tu (Spot c) and transaldolase (Spot S5) were also identified by the comparative secretomic analysis of the 12 *V. parahaemolyticus* isolates in this study. The EF-Tu (Spot c) secreted by all the *V. parahaemolyticus* isolates was reported to be exposed on the cell surface of *Streptococcus*, *Neisseria*, and *Mycoplasma* and under certain conditions on *E. coli* (Mikhalchik et al., 2019). The transaldolase (Spot S5) was secreted by the *V. parahaemolyticus* CHN-B2-28, CHN-B6-62, CHN-B8-26, CHN-N4-18, CHN-N10-18, CHN-L7-40, and CHN-Q5-1 isolates. Gonzalez-Rodriguez et al. reported that the transaldolase (Spot S5) recruited on the cell surface via a non-classical secretion mechanism or an uncharacterized translocation pathway and acted as an important colonization factor for the survival of *Bifidobacterium bifidum* in host intestinal tract (Gonzalez-Rodriguez et al., 2012). In this study, some other extracellular proteins involved in bacterial virulence were also identified. For instance, the superoxide dismutase (Spot S26) secreted by the *V. parahaemolyticus* CHN-B5-29 and CHN-B6-62 isolates is an important virulence factor of *Vibrio alginolyticus* and contributes to the antioxidative stress with potential application for live attenuated vaccine (Chen et al., 2019).

The initial contact and anchoring of bacteria to a host cell are essential during the process of infection (Li et al., 2019). Adhesions are present at the bacterial cell surface or released into extracellular space to form a contact platform for bacterial attachment onto a host cell (Zhang and Orth, 2013). In this study, our secretomic data derived from the 12 *V. parahaemolyticus* isolates revealed such proteins involved in the adhesions of pathogenic bacteria. For example, the TolC (Spot a), secreted by all the *V. parahaemolyticus* isolates, is a major adhesin in *V. harveyi* (Zhu et al., 2019). The enolase (Spot S2), secreted by the *V. parahaemolyticus* CHN-B2-28, CHN-B5-29, CHN-B6-62, CHN-N1-56, CHN-N3-2, CHN-N4-18, CHN-N8-5, and CHN-Q5-1 isolates, is an adhesion-related factor that binds plasminogen and allows bacteria to acquire surface-associated proteolytic activity that facilitates invasion and dissemination in the infected host (Jiang et al., 2014). Bacterial membrane proteins can act as adhesion factors or adhesion enhancers (Gordon et al., 2015). For example, the GAPDH (Spot S13), like many housekeeping proteins, has been presumed to exist only in the cytoplasm to involve in glycolysis. However, it has been reported that GAPDH can be recruited on the cell surface and secreted via a non-classical secretion mechanism, and therefore it is a suitable vaccine candidate for protection against bacterial and parasitic diseases (Perez-Casal and Potter, 2016). The nitrogen regulatory protein P-II (Spot S28) is one of the most widely distributed families of signal transduction proteins widespread among bacteria, archaea, and plants (Radchenko and Merrick, 2011) and control the activities of a very diverse range of enzymes, transcription factors and some membrane transport proteins by direct interaction with their target hosts (Merrick, 2014). The maltoporin (Spot b), secreted by all the *V. parahaemolyticus* isolates, belongs to the outer membrane porin family of Gram-negative bacteria (Thoma et al., 2017) and is a versatile vaccine candidate in *Vibrio* species (Lun et al., 2014). Recently, Yang B. et al. (2019) reported that the maltoporin (Spot b) also contributed to the adhesion and invasion ability of *Aeromonas veronii* TH0426 to epithelioma papulosum cyprini cells. Additionally, bacterial flagellins, potent immunomodulatory agents, contribute to bacterial adhesion and invasion of host cells as well (Hajam et al., 2017). In this study, the flagellin C (Spot S7) was secreted by the *V. parahaemolyticus* CHN-B2-28, CHN-B8-26, CHN-N4-18, CHN-L7-40, and CHN-Q5-1 isolates, whereas the polar flagellin B/D (Spot S8) was secreted by the CHN- B2-28 and CHN-B8-26 isolates. These identified extracellular proteins could be the main targets of vaccine development because of their exposed epitopes on the cell surface.

In this study, comparative proteomic analysis also revealed several intracellular proteins related to bacterial virulence, including the AdhE (Spot P1), OmpAs (Spot P15 and Spot P78), AhpC (Spot P23), PtsA (Spot P36), and PGK (Spot P95). For instance, the AdhE (Spot P1) was expressed by the majority of *V. parahaemolyticus* strains (except the CHN-B8-26, N2-5, N8-5, and L7-40 isolates). Luong et al. (2015) reported that Δ*adhE* mutant strain decreased pneumolysin (Ply) under ethanol stress condition when compared to wild-type strain and implied that AdhE was a pneumococcal virulence factor in *Streptococcus pneumoniae*. The OmpAs (Spot P15 and Spot P78), expressed by the *V. parahaemolyticus* CHN-B2-28, CHN-B5-29, CHN-N1-56, CHN-N4-18, and CHN-Q5-1 isolates, belong to a group of surface-exposed porins associated with bacterial pathogenesis in *V. parahaemolyticus* (Cheng et al., 2018). In this study, the AhpC (Spot P23) was expressed by the CHN-B2-28, CHN-N1-56, CHN-N3-2, CHN-N4-18, CHN-N10-23, and CHN-Q5-1 isolates. It has been reported that AhpC in highly virulent *Francisella tularensis* serves as a key antioxidant enzyme and contributes to its robust oxidative and nitrosative stress resistance and intramacrophage survival and consequently serves as a virulence factor (Alharbi et al., 2019). The PtsA (Spot P36), expressed by the CHN-B5-29, CHN-B6-62, CHN-N2-5, CHN-N3-2, CHN-N4-18, and CHN-L7-40 isolates, is an intracellular protein of the monosaccharide phosphotransferase systems and also localizes to the bacterial cell wall as an adhesin in *S. pneumoniae* (Mizrachi Nebenzahl et al., 2016). The PGK (Spot P95), expressed by the CHN-Q5-1 isolate from *C. idellus*, is a key enzyme of glycolysis and also acts as a mediator of DNA replication and repair in the nucleus (Kumar et al., 2019). This protein, also identified in our prior research (He et al., 2015), has been used as an antigen in a neonatal-animal model against *Streptococcus agalactiae* infection (Wang et al., 2014).

The increase in MDR pathogenic bacteria has raised a serious public health and economic concern (Elmahdi et al., 2016). One common mechanism for bacteria to obtain antibiotic resistance is to actively pump drugs from bacterial cells by employing ABC transporters (Wilson, 2016). The functions of ABC transporters are very diverse, ranging from importing essential nutrients to conferring drugs in bacteria, archaea, and eukaryote (Beis, 2015). In this study, comparative secretomic analysis revealed several ABC transporters of the *V. parahaemolyticus* isolates with resistance phenotypes. For instance, the maltodextrin-binding protein (Spot d), secreted by all the *V. parahaemolyticus* isolates, is part of the maltose ABC complex MalEFGK (Machtel et al., 2019). The D-ribose ABC TSBP (Spot S15) and arginine ABC TSBP (Spot S18) were secreted by most of the isolates, except the CHN-B6-62 and CHN-Q5-1, as well as CHN-B6-62 and CHN-N1-56 isolates, respectively. The choline ABC TSBP (Spot S14) was secreted by the CHN-B2-28, CHN-B5-29, CHN-N1-56, CHN-N2-5, and CHN-N8-5 isolates. The glycine/betaine ABC TSBP (Spot S12) was observed only on the secretomic profile derived from the CHN-B2-28 and CHN-B8-26 isolates, whereas the peptide ABC TPPBP (Spot S24) was observed from the CHN-B2-28 and CHN-N2-5 isolates. The other possible mechanism of bacterial resistance is the ribosome protection (Wilson, 2016). Bacterial ribosome, being one of the main antibiotic targets in bacterial cells (Wilson, 2014), is a large protein–RNA complex that consists of two major subunits (a small 30S subunit and a large 50S subunit), each of which is composed of a variety of proteins. In this study, two translation-associated proteins EF-Tu (Spot c) and EF-Ts (Spot S22) were identified by the secretomic and proteomic analysis. The former existed in all the *V. parahaemolyticus* isolates, whereas the latter was secreted by the CHN-B2-28, CHN-B8-26, CHN-N3-2, CHN-N4-18, CHN-N10-18, CHN-L7-40, and CHN-Q5-1 isolates. The EF-Ts is a guanosine nucleotide exchange factor for EF-Tu (He et al., 2015), and the EF-Tu catalyzes the binding of aminoacyl-tRNA (aa-tRNA) to a site of the ribosome during protein synthesis (Daviter et al., 2003). Agarwal and O’Connor (2014) reported that a ribosomal protein S12 binding the EF-Tu contributed to streptomycin resistance in *E. coli* MC323. Overall, these identified proteins may serve as an explanation for the resistance phenotypes of the *V. parahaemolyticus* isolates with aquatic product origins.

In this study, all the *V. parahaemolyticus* isolates (except the CHN-B6-62 isolate) produced more intracellular protein spots on their proteomic profiles responding to aquatic product matrices. A total of 71 differential intracellular proteins were identified by the LC-MS/MS analysis, most of which were involved in biosynthesis process (e.g., diaminopimelate, lysine, ADP-L-glycero-β-D-manno-heptose, *S*-adenosylmethionine, GMP, riboflavin, and fatty acid biosynthesis), metabolic processes (e.g., gluconeogenesis, glycerol, one-carbon, glycerol-3-phosphate, alcohol, carbohydrate, D-ribose, deoxyribonucleotide, and amino acid metabolism), and cell membrane composition. The growth of microorganism may vary with the available carbon and nitrogen sources (Chiang and Chou, 2008). In this study, our comparative proteomic analysis showed that the crustacean matrices changed the proteomes of the *V. parahaemolyticus* CHN-N1-56 and CHN-N2-5 isolates, recovered from *L. vannamei* and *O. oratoria*, respectively, more than the other 10 types of aquatic product matrices. Wang et al. (2018) evaluated the influence of food matrices (shrimp, oyster, freshwater fish, pork, chicken, and egg fried rice) on extracellular products of *V. parahaemolyticus* and found that *V. parahaemolyticus* expressed significantly higher activity (*p* < 0.05) of gelatinase, caseinase, urease, DNase, and amylase in shrimp matrix than freshwater fish. In this study, our data also showed that the crude fat contents of the crustacean matrices were approximately 101- to 85-fold higher than those of the fish and shellfish matrices. Lipases expressed by *Vibrio* species can hydrolyze fats into glycerol and fatty acids (Beshiru and Igbinosa, 2018). Moravec et al. (2017) reported the *Vibrio*’s ability to acquire fatty acids from environmental sources. Exogenous fatty acids can affect bacterial metabolism, modification of membrane lipids, alteration of protein function, regulation of gene expression, and stress responses (Moravec et al., 2017; Yao and Rock, 2017). In this study, our comparative proteomic data highlighted the significance of monitoring *V. parahaemolyticus* contamination in fat-rich aquatic products in the future research.

In this study, the other interesting finding was that some *V. parahaemolyticus* isolates produced virulence-associated proteins when incubated only in aquatic product matrices media. For instance, the OmpA family proteins (Spots D2-1, D2-2, D2-3, H2-1, and L2-2) were expressed by the *V. parahaemolyticus* CHN-B8-26 and CHN-N10-18 isolates when grown in the *S. strictus* and *O. gigas thunberg* matrices media, respectively. Recent research has indicated that proteins and lipids can form complexes (called liprotides) that assist in folding of outer membrane proteins, for example, OmpA, especially optimal folding at pH 8–9 (Nedergaard Pedersen et al., 2018). Protein folding is the essential process by which a polypeptide chain acquires its functional, native 3D structure. In this study, our data suggested that the aquatic product matrices with higher contents of protein and fat may facilitate the expression of virulence-associated factors (e.g., the OmpA family proteins) in the *V. parahaemolyticus* isolates.

Although gel-based proteomes are laboring and time-consuming, images of 2D-GE gels can be compared so as to quantify each protein spot from different samples, and these protein spots can subsequently be excised, sequenced, and identified with MS, especially LC-MS/MS (Lee et al., 2019). Each protein has multiple forms as a result of genetic variations, splicing, truncation, degradation, and posttranslational modifications; therefore, innovated proteomic technologies such as MS-based shotgun proteomics (Lee et al., 2018) should be employed to explore more virulence and resistance-associated factors in *V. parahaemolyticus*. Also, new proteins during culture handing/extraction processing should be prevented by adding a compound (e.g., chloramphenicol), and the function of the virulence and resistance-related proteins identified in the *V. parahaemolyticus* isolates should be further pursued by cell and animal infection mode analysis in the future research.

## Data Availability Statement

The datasets generated for this study are available on request to the corresponding author.

## Author Contributions

ZZ, LY, PY, YW, XP, and LC participated in the design and discussion of the study. ZZ carried out the experiments. LY performed the qRT-PCR assay. ZZ and LC wrote the manuscript. All the authors read and approved the final version to be published.

## Conflict of Interest

The authors declare that the research was conducted in the absence of any commercial or financial relationships that could be construed as a potential conflict of interest.

## Footnotes

1 http://go.princeton.edu/cgi-bin/GOTermFinder

2 http://www.premierbiosoft.com/

## Supplementary Material

FIGURE S1 The secretomic profiles of the *V. parahaemolyticus* ATCC33847 and ATCC17802 strains by the 2D-GE analysis. **(A)** ATCC33847. **(B)** ATCC17802. The protein spots marked with blue circles and the numbers in red were the same at similar locations on the secretomic profiles of the 12 *V. parahaemolyticus* isolates. pI, isoelectric point.

FIGURE S2 The GO functional classification of the differential intracellular proteins. **(A)** The differential intracellular proteins of 12 *V. parahaemolyticus* isolates. **(B)** The differential intracellular proteins of the 12 *V. parahaemolyticus* isolates incubated between in the TSB and aquatic product matrix media.

FIGURE S3 The proteomic profiles of the *V. parahaemolyticus* ATCC33847 and ATCC17802 strains. **(A)** ATCC33847. **(B)** ATCC17802. The intracellular protein spots marked with the numbers in red were the same at similar locations on the proteomic profiles of the 12 *V. parahaemolyticus* isolates.

FIGURE S4 The expression of several representative genes encoding differential proteins by the RT-PCR assay.

FIGURE S5 The protein, carbohydrate and fat contents of the 12 types of aquatic product matrices.

TABLE S1 Oligonucleotide primers used for the RT-PCR assay in this study.
