# Supplementary figures and images for: Comparative Proteomics and Secretomics Revealed Virulence and Antibiotic Resistance-Associated Factors in Vibrio parahaemolyticus Recovered From Commonly Consumed Aquatic Products
Source: Front Microbiol. 2020 Jul 14;11:1453. doi: 10.3389/fmicb.2020.01453 (PMC7381183; doi:10.3389/fmicb.2020.01453)

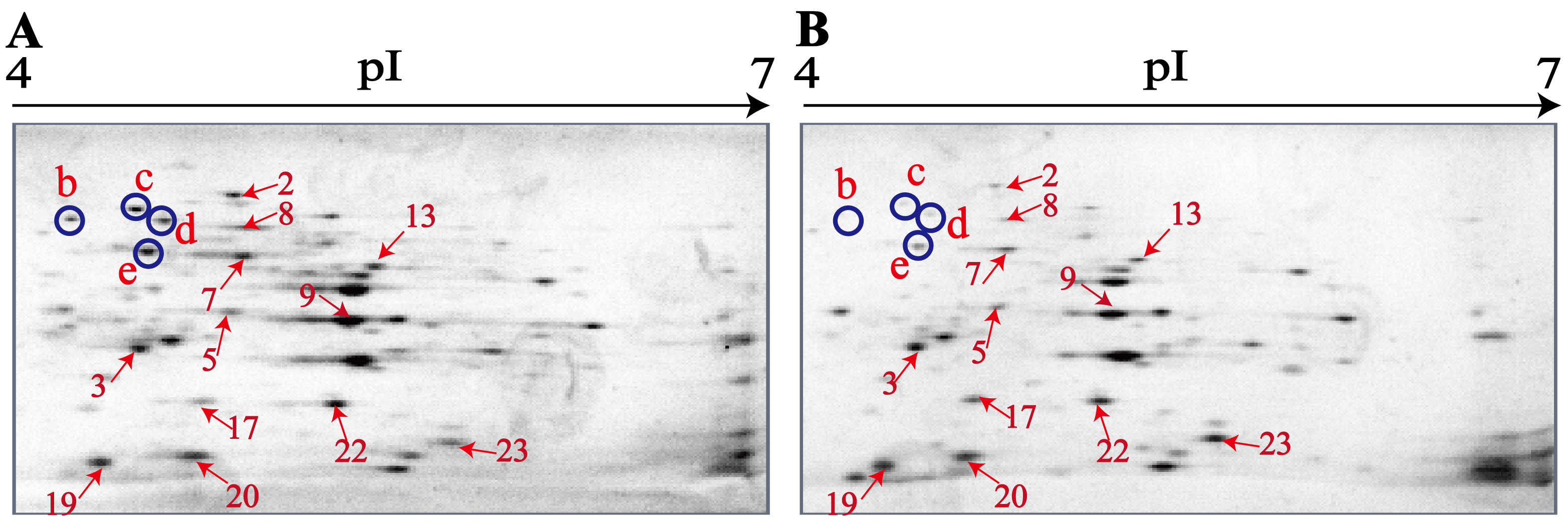

Supplement: FIGURE S1 — The secretomic profiles of the V. parahaemolyticus ATCC33847 and ATCC17802 strains by the 2D-GE analysis. (A) ATCC33847. (B) ATCC17802. The protein spots marked with blue circles and the numbers in red were the same at similar locations on the secretomic profiles of the 12 V. parahaemolyticus isolates. pI, isoelectric point. [file Image_1.TIF]

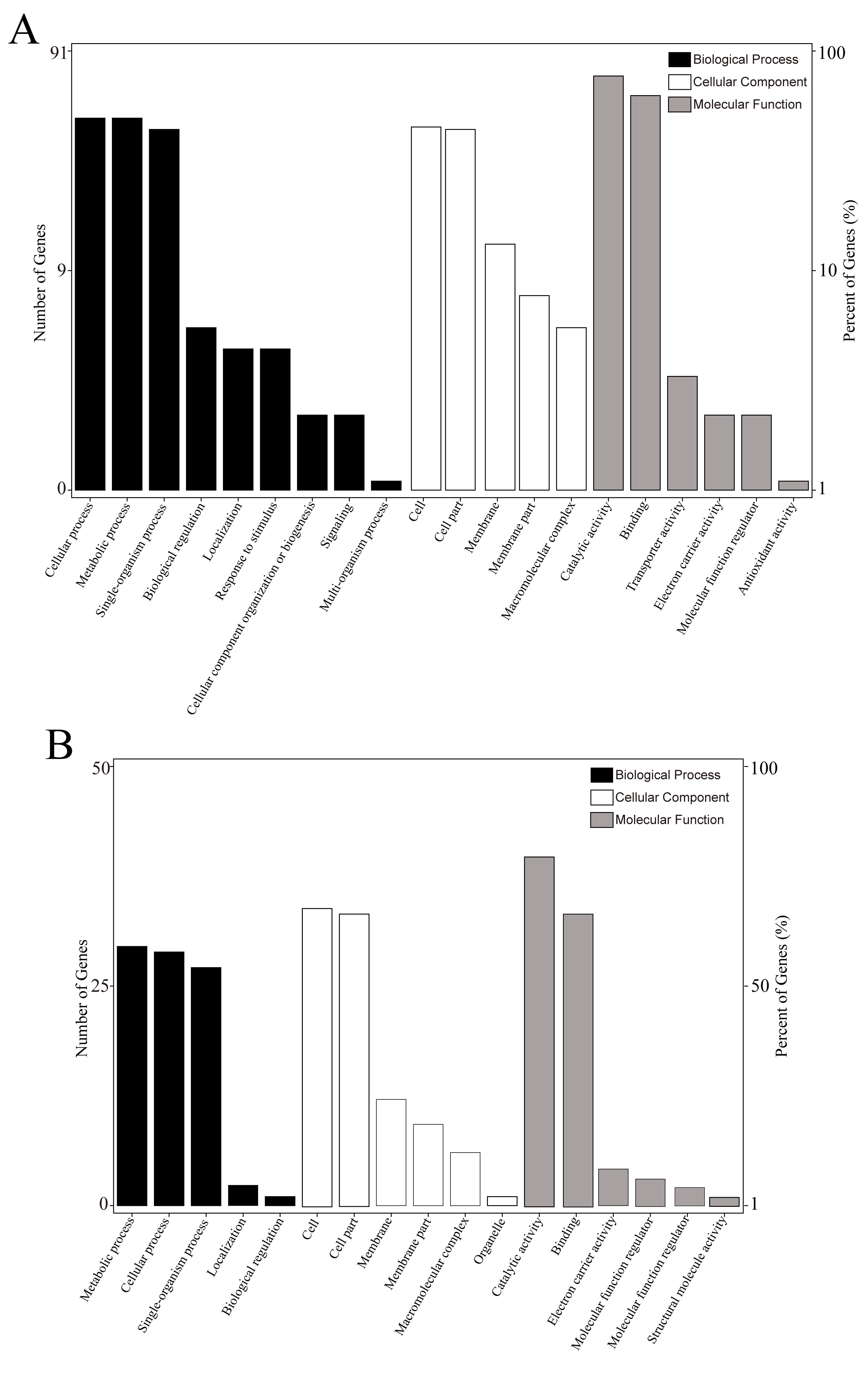

Supplement: FIGURE S2 — The GO functional classification of the differential intracellular proteins. (A) The differential intracellular proteins of 12 V. parahaemolyticus isolates. (B) The differential intracellular proteins of the 12 V. parahaemolyticus isolates incubated between in the TSB and aquatic product matrix media. [file Image_2.TIF]

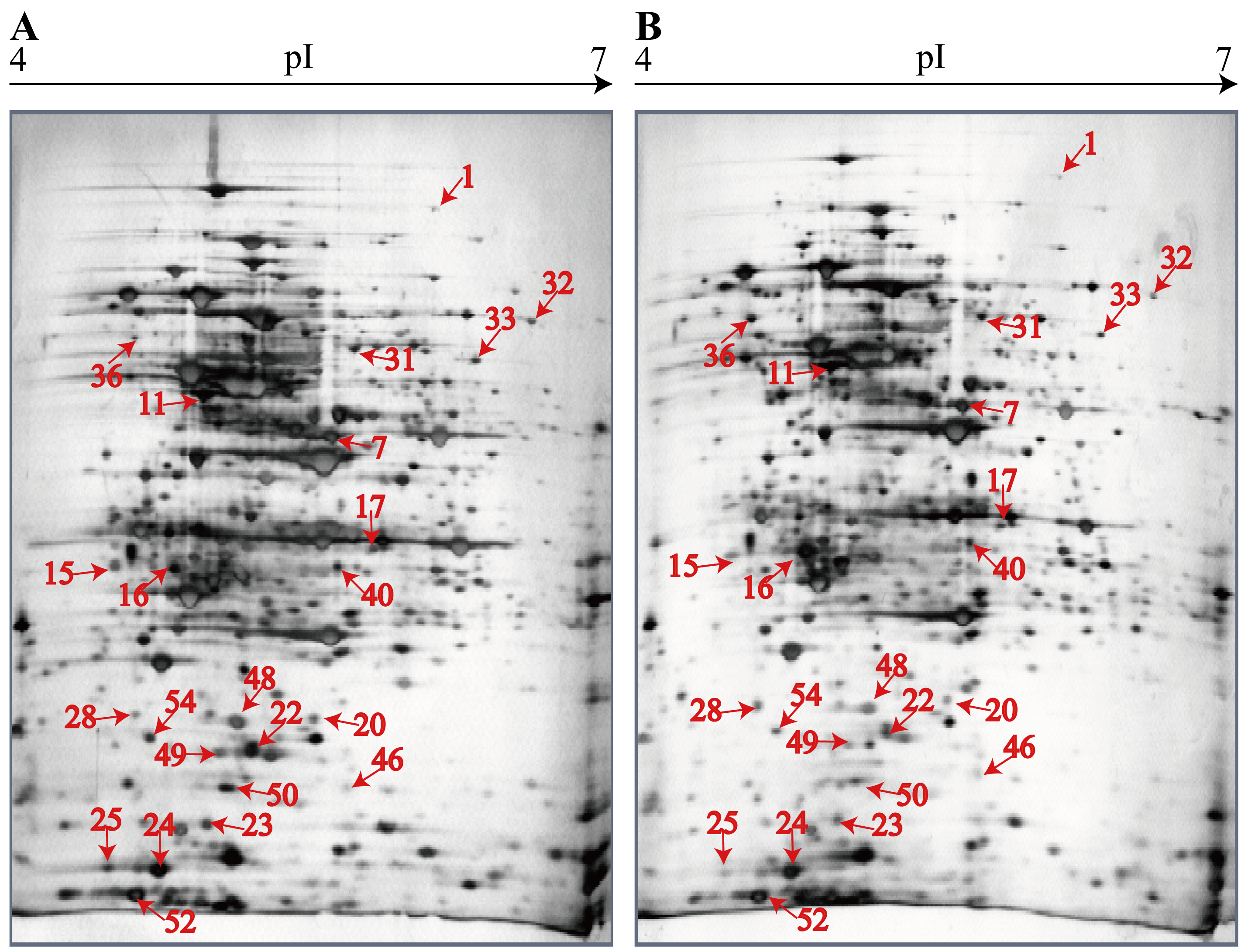

Supplement: FIGURE S3 — The proteomic profiles of the V. parahaemolyticus ATCC33847 and ATCC17802 strains. (A) ATCC33847. (B) ATCC17802. The intracellular protein spots marked with the numbers in red were the same at similar locations on the proteomic profiles of the 12 V. parahaemolyticus isolates. [file Image_3.TIF]

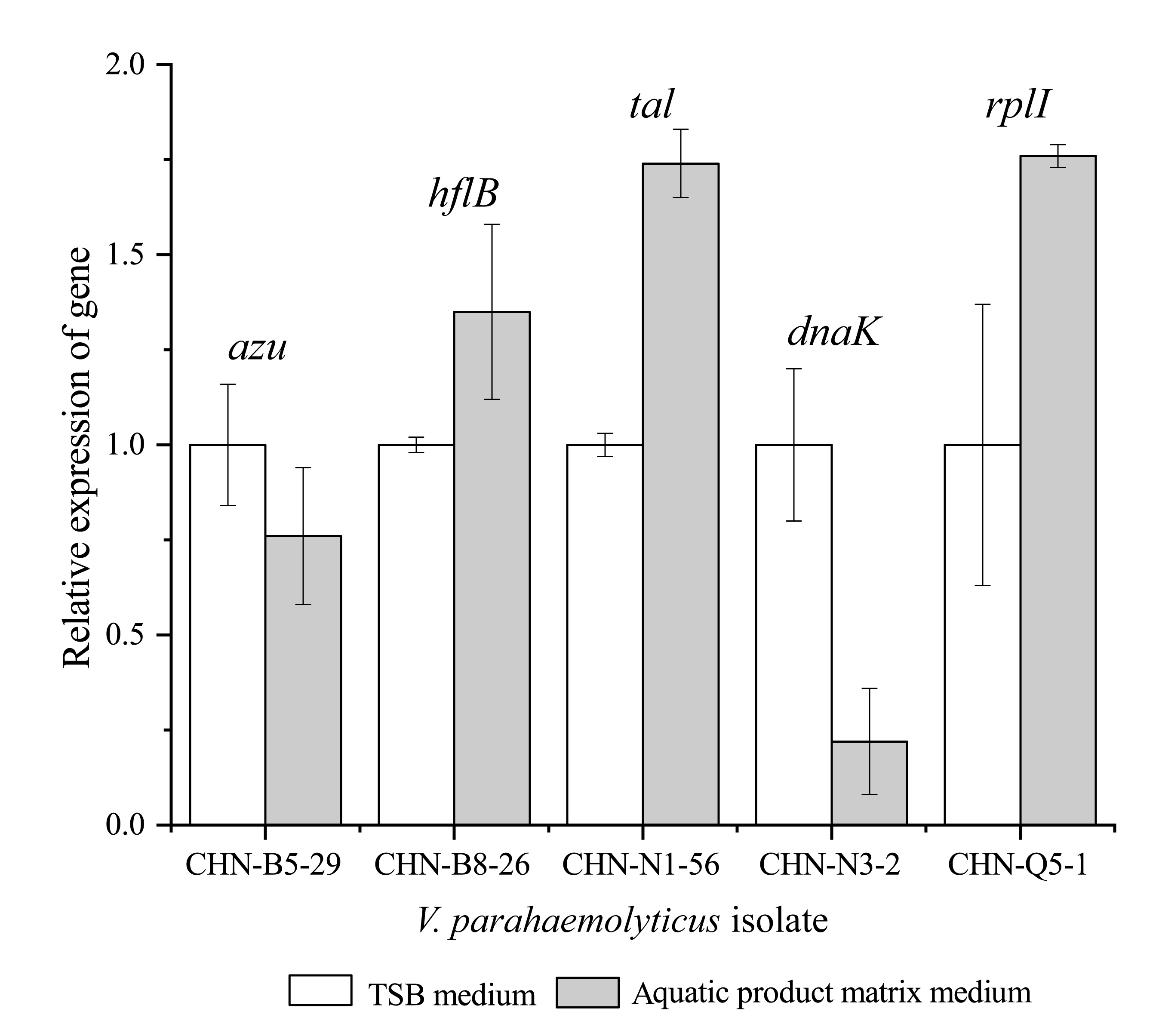

Supplement: FIGURE S4 — The expression of several representative genes encoding differential proteins by the RT-PCR assay. [file Image_4.TIF]

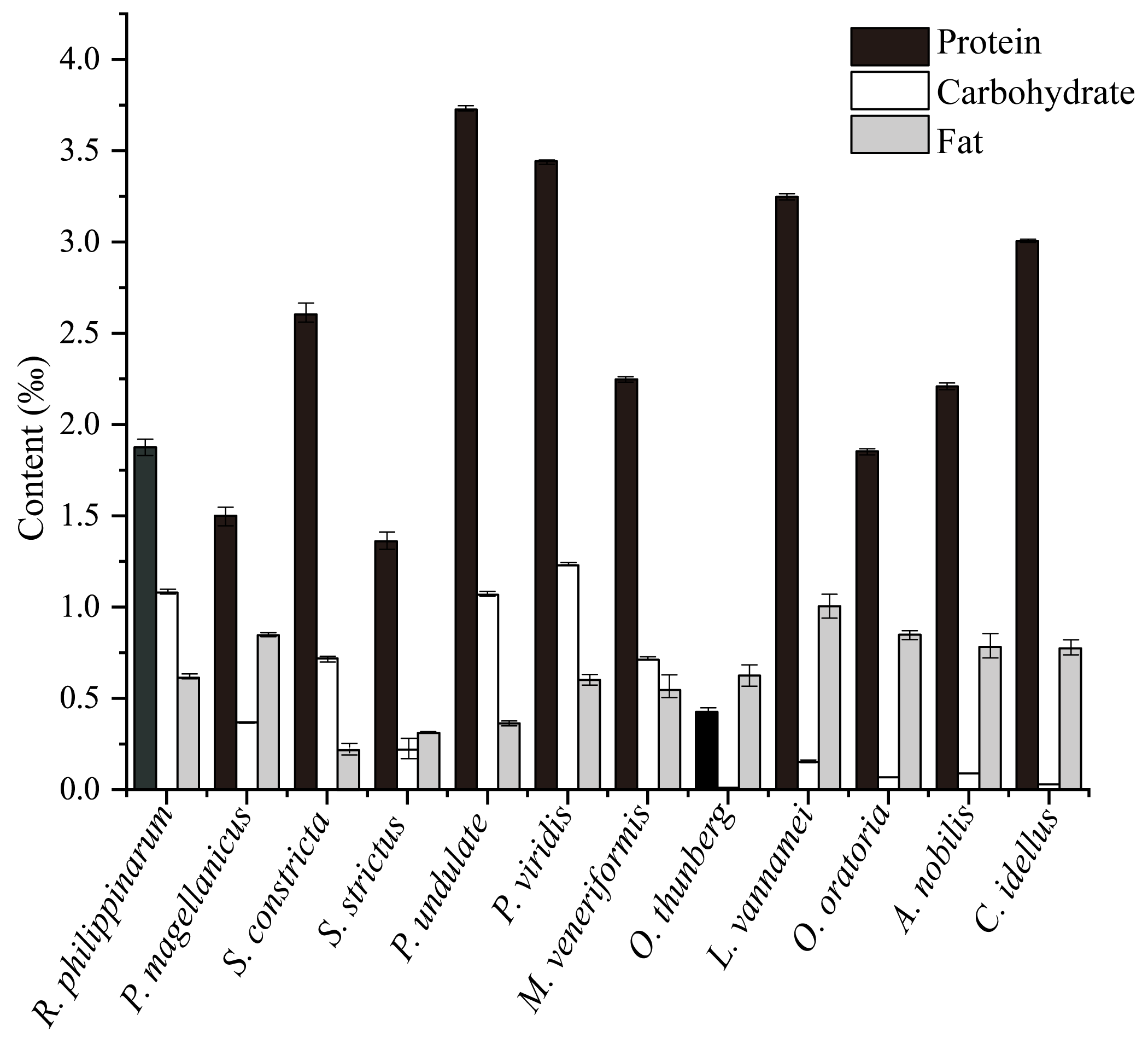

Supplement: FIGURE S5 — The protein, carbohydrate and fat contents of the 12 types of aquatic product matrices. [file Image_5.TIF]
