# Supplementary material for: Comparative Proteomics and Secretomics Revealed Virulence and Antibiotic Resistance-Associated Factors in Vibrio parahaemolyticus Recovered From Commonly Consumed Aquatic Products
Source: Front Microbiol. 2020 Jul 14;11:1453. doi: 10.3389/fmicb.2020.01453 (PMC7381183; doi:10.3389/fmicb.2020.01453)
Supplement: TABLE S1 — Oligonucleotide primers used for the RT-PCR assay in this study. [file Table_1.docx]

**TABLE S1 Oligonucleotide primers used for the RT-PCR assay in this study**

| **Gene** | **Description of encoded protein** | **Primer** | **Sequence (5 to 3)** | **Predicted amplicon size (bp)** |
| --- | --- | --- | --- | --- |
| *azu* | Azurin | *azu*-F | ATGTCTGCTGGCGCAGATAACAGTT | 140 |
|  |  | *azu*-R | CCAGTGACCAGGGAATGAGC |  |
| *hflB* | ATP-dependent metallopeptidase HflB | *hflB*-F | TTCAGGACCGTAAATTAGCT | 102 |
|  |  | *hflB*-R | CCAGAACAAGACCGAGTAAG |  |
| *dnaK* | Chaperone protein DnaK | *dnaK*-F | TCATTACGCCACCCATAGTCT | 177 |
|  |  | *dnaK*-R | AAGCGAAAGTTGCTGAGTTCTT |  |
| *tal* | Transaldolase | *tal*-F | CAGGAGCGATAGTTAGACGG | 184 |
|  |  | *tal*-R | GGTACAAAGCGAAAGAAGG |  |
| *rplI* | 50S ribosomal protein L9 | *rplI*-F | CTGGTGACGAAGGCAAACTA | 199 |
|  |  | *rplI*-R | TCAGCAGCAACAACCTGTAC |  |
| *16S rRNA* |  | *16S rRNA* -F | GACACGGTCCAGACTCCTAC | 179 |
|  |  | *16S rRNA* -R | GGTGCTTCTTCTGTCGCTAAC |  |
